# pH-Dependent Solution Micellar Structure of Amphoteric Polypeptoid Block Copolymers with Positionally Controlled Ionizable Sites

**DOI:** 10.1021/acs.biomac.3c00407

**Published:** 2023-07-21

**Authors:** Meng Zhang, Yun Liu, Xiaobing Zuo, Shuo Qian, Sai Venkatesh Pingali, Richard E. Gillilan, Qingqiu Huang, Donghui Zhang

**Affiliations:** † Department of Chemistry and Macromolecular Studies Group, Louisiana State University, Baton Rouge, Louisiana 70803, United States; ‡ Center for Neutron Research, National Institute of Standards and Technology, Gaithersburg, Maryland 20899, United States; § X-ray Science Division, Argonne National Laboratory, Lemont, Illinois 60439, United States; ∥ Neutron Scattering Division and Second Target Station, Oak Ridge National Laboratory, Oak Ridge, Tennessee 37831, United States; ⊥ Neutron Scattering Division, Oak Ridge National Laboratory, Oak Ridge, Tennessee 37831, United States; # MacCHESS (Macromolecular Diffraction Facility at CHESS), Cornell University, Ithaca, New York 14850, United States

## Abstract

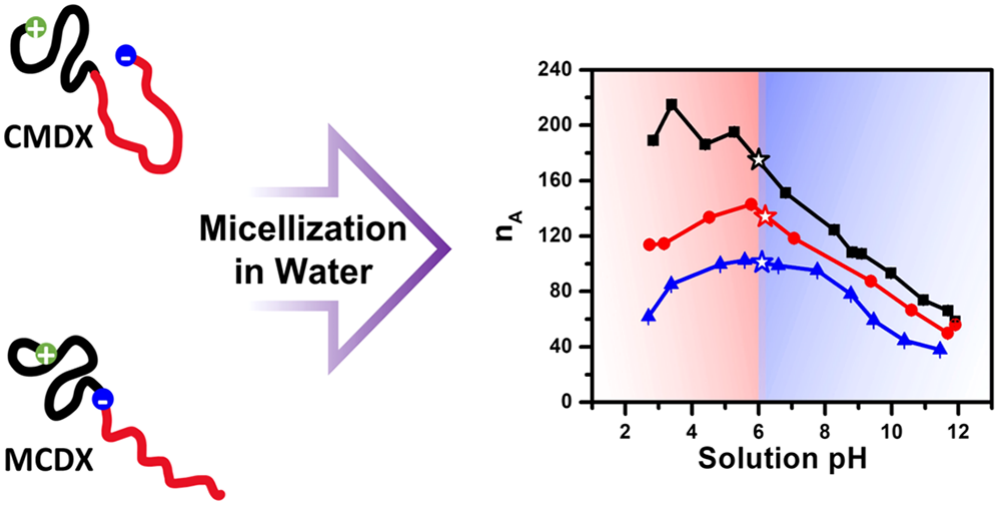

While solution micellization of ionic block copolymers (BCP) with randomly distributed ionization sites along the hydrophilic segments has been extensively studied, the roles of positionally controlled ionization sites along the BCP chains in their micellization and resulting micellar structure remain comparatively less understood. Herein, three amphoteric polypeptoid block copolymers carrying two oppositely charged ionizable sites, with one fixed at the hydrophobic terminus and the other varyingly positioned along the hydrophilic segment, have been synthesized by sequential ring-opening polymerization method. The presence of the ionizable site at the hydrophobic segment terminus is expected to promote polymer association toward equilibrium micellar structures in an aqueous solution. The concurrent presence of oppositely charged ionizable sites on the polymer chains allows the polymer association to be electrostatically modulated in a broad pH range (ca. 2–12). Micellization of the amphoteric polypeptoid BCP in dilute aqueous solution and the resulting micellar structure at different solution pHs was investigated by a combination of scattering and microscopic methods. Negative-stain transmission-electron microscopy (TEM), small-angle neutron scattering (SANS), and small-angle X-ray scattering (SAXS) analyses revealed the dominant presence of core–shell-type spherical micelles and occasional rod-like micelles with liquid crystalline (LC) domains in the micellar core. The micellar structures (e.g., aggregation number, radius of gyration, chain packing in the micelle) were found to be dependent on the solution pH and the position of the ionizable site along the chain. This study has highlighted the potential of controlling the position of ionizable sites along the BCP polymer to modulate the electrostatic and LC interactions, thus tailoring the micellar structure at different solution pH values in water.

## Introduction

Electrostatic interactions encoded in the monomer sequence, a.k.a. charge pattern, are known to play important roles in the structure and function of many biomacromolecules (e.g., polysaccharides, intrinsically disordered proteins, or protein regions).^1−4^ Understanding and manipulating charge pattern to control the chain conformation and collective assembly of synthetic polymers is still at its infancy despite the significant potential to fine-tune the material properties of these polymer assemblies.^5−8^ Several recent studies have highlighted the role of charge pattern in modulating the micellar structure^9−11^ or the solution phase behavior of sequence-controlled synthetic polymers/oligomers.^12,13^ Given the growing synthetic capabilities that allow for control over monomer sequence in a wide range of polymeric and oligomeric chains,^14−17^ there is an impetus to develop a better understanding regarding the role of charge pattern in the solution assembly and structure of synthetic polymers.

Ionic block copolymers (BCPs), in selective solvents, can self-assemble to form equilibrium micellar structures with spherical, cylindrical, and lamellar geometry governed by the minimization of the free energy contributions from the core segment, corona segment, and core–corona interface.^18^ Nonequilibrium assembly of ionic BCP micelles can afford a diverse range of intermediate morphologies that are kinetically trapped.^19^ Electrostatic interactions are known to play an important role in the micellization and micellar structure of ionic BCPs, giving rise to their structural dependence on the solution pH,^20−22^ ionic strength,^20−23^ and specific ions in aqueous media.^24,25^ As a result, ionic BCP micelles are used in a wide range of technical applications including protein encapsulation and delivery,^26,27^ ion transport media,^28^ template synthesis,^29^ etc. Early studies of ionic BCP micelles have mainly focused on those where the ionizable monomers are randomly distributed in the solvophilic segments, installed by postpolymerization functionalization approach.^30^ Incorporation of ionizable monomers into the solvophobic segments of ionic BCPs has been comparatively less explored. Several studies have shown that incorporating ionizable monomers to the solvophobic segment of ionic BCPs can enhance the chain mobility in aqueous solution, thereby facilitating the formation of equilibrium micelles.^31−33^ In addition, positioning of an ionic monomer at the solvophobic chain ends has been shown to induce looplike conformation of solvophobic core segment of ionic BCP micelles due to the strong propensity of the ionic monomer to be solvated by the polar organic solvent.^34−37^

Polypeptoid polymers featuring *N*-substituted polyglycine backbone has emerged as an intriguing class of peptidomimetic polymers.^38−40^ Due to *N*-substitutions, polypeptoids have more flexible backbones with a reduced propensity to form secondary structures and lack extensive hydrogen bonding along the backbone relative to polypeptides. In addition, polypeptoids exhibit good cytocompatibility, enhanced proteolytic stability, and processability relative to their polypeptide counterparts.^41−43^ As a result, the solution nanostructures comprised of polypeptoid polymers with controlled geometry and dimension have been increasingly investigated as materials candidates for various biotechnological applications (e.g., drug delivery carrier, theragnostic agent, tissue engineering matrices).^44−46^ Polypeptoids with precisely defined monomer sequences and discrete chain lengths can be synthesized by the submonomer method.^47^ This stepwise approach is limited in the accessible chain length (typically degree of polymerization <50) and synthetic scalability. By contrast, controlled ring-opening polymerizations of *N*-substituted glycine derived *N*-carboxyanhydride have been developed to enable access to well-defined polypeptoid BCPs with long average chain lengths and controlled block sequences, notwithstanding the inherent chain length and compositional dispersity.^39,48^ Our earlier studies of sequence-defined peptoid diblock copolymers with a discrete chain length have revealed that the position and number of charged monomers along the chain can modulate the aggregation number and size of spherical micelles in aqueous solution in a highly predictable manner.^9,11^ Separately, the coupled charge and aromatic residue pattern has been shown to influence the chain conformation and stability of sequence-defined peptoid micelles with lamellar or spherical geometry.^49^ In comparison, it remains ambiguous regarding whether the charge pattern encoded in the block sequences of ionic BCP obtained by controlled polymerization methods can effectively modulate the polymer association to form micelles with distinct structural characteristics, considering the statistical variation of chain length and composition inherent to these BCP that may smear the effect of the charge pattern.

In this contribution, we designed and synthesized three amphoteric polypeptoid block copolymers with controlled ionizable sites along the chains by sequentially controlled ring-opening polymerizations and investigated their micellization and pH-dependent micellar structure in aqueous solution by a combination of transmission electron microscopy (TEM) and light scattering/small-angle X-ray scattering (SAXS)/small-angle neutron scattering (SANS) techniques. The polypeptoid BCPs in this study contain two oppositely charged ionizable sites along the chain: the cationic site is fixed at the hydrophobic segment terminus, whereas the location of the anionic site was systematically varied along the chain. Three strategic locations were selected for the anionic site: at the hydrophilic segment terminus, at the junction of the hydrophilic and hydrophobic segments, and randomly distributed within the hydrophilic segment. The presence of the cationic site at the hydrophobic segment terminus is expected to promote polymer association toward equilibrium micellar structures in aqueous solution. The concurrent presence of both cationic and anionic sites on the polymer chains allows the polymer association to be electrostatically modulated in a broad pH range (ca. 2–12). We have found that the position of the ionizable sites along the polymer chain can modulate the electrostatic interactions and liquid crystalline interaction in the micelles and consequently the pH-dependent micellar structure by altering the aggregation number, micellar size, and polymer chain packing within the micelles.

## Materials and Methods

### General Considerations

All chemicals used were purchased from Sigma-Aldrich,VWR or CIL and used as received, unless further noted. Tetrahydrofuran (THF), hexanes, and dichloromethane (DCM) used in this study were purchased from Sigma-Aldrich and purified by passing through alumina columns under argon gas using a solvent purification system. All *N*-substituted glycine derived *N*-carboxyanhydride monomers were synthesized by the reported procedures.^43,50,51^ All polymerization reactions were conducted under a nitrogen atmosphere in the glovebox. ^1^H NMR spectra were recorded on an AVIII-400 Nanobay spectrometer, and the chemical shifts in parts per million (ppm) were referenced relative to the protio impurities of CDCl_3_.

### Synthesis of Ionic Polypeptoid Block Copolymers

**Scheme 1:**
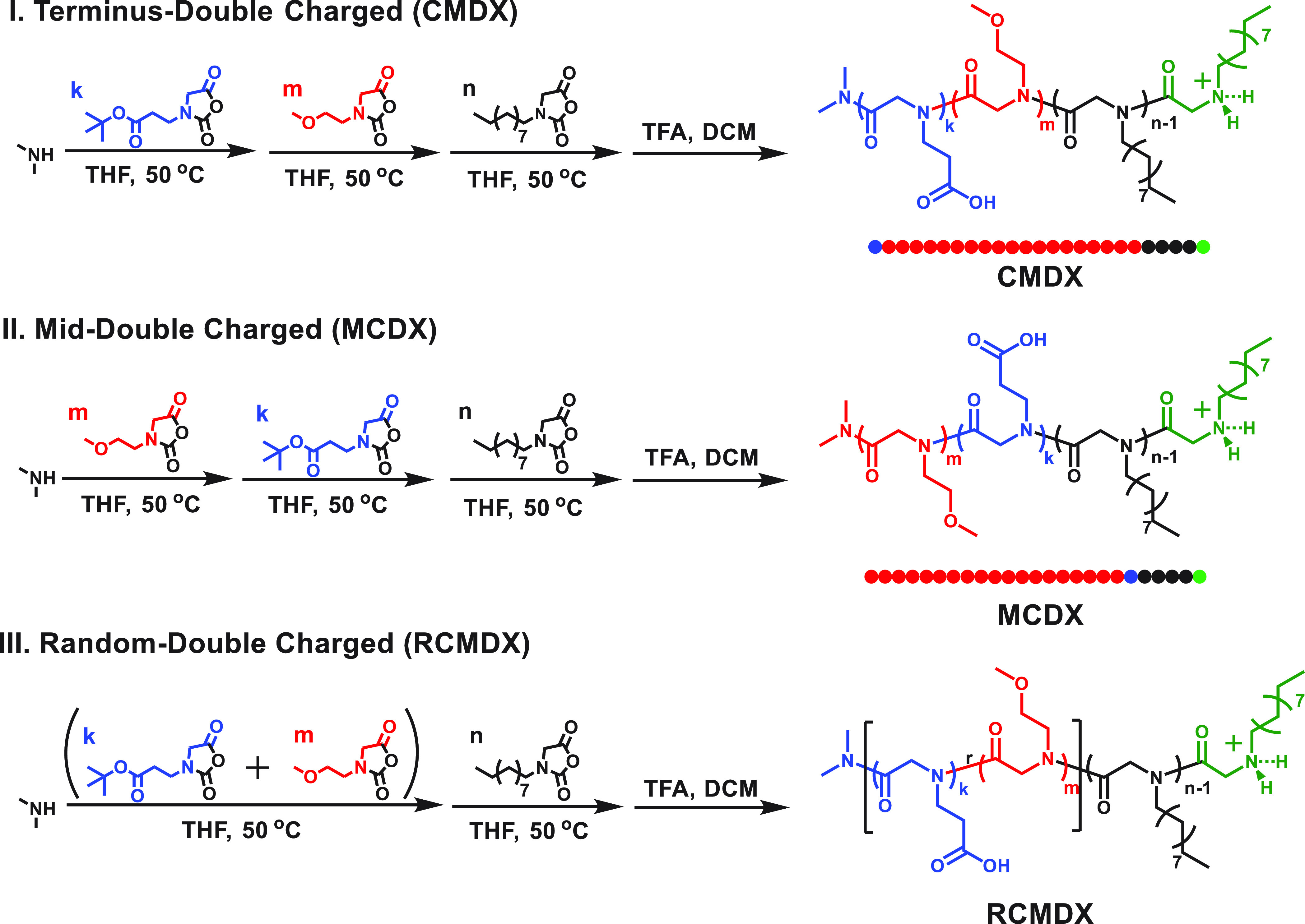


A representative procedure for the synthesis of PNCEtG_1.2_-*b*-PNMeOEtG_17_-*b*-PNDG_5.7_ (CMDX) is given as follows (Scheme 1). Inside the glovebox, ^t^BuCO_2_Et-NCA (M_1_) stock solution in THF (760 μL, 300 μmol, 0.40 M) was added into a 20 mL sealed glass vial containing dimethylamine stock solution in THF (690 μL, 300 μmol, 0.44 M) using a microsyringe. Polymerization of ^t^BuCO_2_Et-NCA ([M_1_]_0_:[I]_0_ = 1:1) proceeded at 50 °C under nitrogen for 45 min to reach complete conversion. MeOEt-NCA (M_2_) stock solution (11.9 mL, 5.8 mmol, 0.49 M) was then added to the above reaction mixture. Polymerization of MeOEt-NCA ([M_2_]_0_:[I]_0_ = 19:1) proceeded at 50 °C for another 52 h to reach complete conversion. De-NCA (M_3_) stock solution in THF (3.7 mL, 1.5 mmol, 0.42 M) was subsequently added to the above reaction mixture. Polymerization of De-NCA proceeded at 50 °C for an additional 46 h to reach completed conversion. Between each polymerization, an aliquot (10 μL) of the reaction mixture was taken to confirm the quantitative conversion of the monomers by FT-IR spectroscopy (Figure S1). Upon completion of the three polymerization steps, the volatiles were removed under a vacuum to yield a sticky polymer. The crude polymer was then redissolved in CDCl_3_ (2.25 mL) and TFA (0.75 mL) and stirred at room temperature overnight. The volatiles were removed under vacuum to afford a light-yellow gel, which was further purified by dialysis in water followed by lyophilization to afford a white fluffy powder (0.67 g, 66% yield). ^1^H NMR spectra of CMDX, MCDX and RCMDX block copolymers, and the respective precursors before TFA treatment are shown in Figures S2–S7.

### Preparation of Ionic Block Copolymer Solutions

Solutions of PNCEtG-*b*-PNMeOEtG-*b*-PNDG (CMDX), PNMeOEtG-*b*-PNCEtG-*b*-PNDG (MCDX) and P(NCEtG-*r*-NMeOEtG)-*b*-PNDG (RCMDX) block copolymers (5.0 mg/mL polymer concentration) were prepared by directly dissolving the corresponding polymers (∼10 mg) in H_2_O or D_2_O (∼2 mL) with 60 mM NaCl solution at room temperature (21 °C) in sealed glass vials and the pH of the solutions were adjusted by adding 0.5 M NaOH or HCl stock solution in H_2_O or D_2_O. The sample solutions were heated up to 80 °C in a metal sample holder for 2 h and slowly cooled back to room temperature (21 °C) overnight to attain the micellar solutions. Then the sample solutions were filtered through polyether sulfone (PES) filters (pore size = 0.45 μm) prior to any further characterization.

### Size-Exclusion Chromatography (SEC) Analyses

SEC analyses of the block copolymers were performed using a Tosoh EcoSEC Model HLC-8230 GPC system (Tosoh Bioscience degasser, isocratic pump, autosampler, and column heater) equipped with a Tosoh Bioscience dual-flow refractive index (RI) detector with a 630–670 nm LED light source and a Tosoh Bioscience LenS3 multiangle light scattering (MALS) detector (30 mW diode laser at λ = 505 nm). The sample columns and reference column were installed with a TSKgel α-M 7.8 mm I.D. × 30 cm, 13 μm packing beads. HFIP with 3 mg/mL CF_3_CO_2_K was used as the eluent at a flow rate of 0.45 mL/min. The column and detector temperatures were set at 40 °C. All data analysis was performed using SECView software. Polymer molecular weight (*M*_n_) and molecular weight distribution (*Đ*) were obtained by analyzing the RALS-dRI data based on the LS and RI instrument constants that were calibrated with a PMMA standard sample (*M*_w_ (LS) = 32350 g/mol, PDI = 1.03) and a set of PMMA standards with various molecular weight at 40 °C. The absolute polymer molecular weight (*M*_n_) was determined using the measured refractive index increment d*n*/d*c* values. The refractive index increment (d*n*/d*c*) of the polymer was determined using a Tosoh Dual-Flow refractive index (RI) detector and a SECView software d*n*/d*c* template. The block copolymer samples were dissolved in HFIP with 3 mg/mL CF_3_CO_2_K to prepare polymer solutions with a known concentration. The solutions were injected into the dRI detector to obtain the polymer molecular weight (*M*_n_) and molecular weight distribution (*Đ*). The SEC-dRI chromatograms of the CMDX, MCDX and RCMDX block copolymers are shown in Figure S8.

### Differential Scanning Calorimetry (DSC) Analyses

DSC analyses of CMDX, MCDX and RCMDX block copolymers were performed on a TA DSC 2920 calorimeter under nitrogen gas. The solid polymer sample (∼4 mg) was sealed in a hermetic aluminum pan, and an empty hermetic aluminum pan was used as a reference. The samples were heated from 10 to 100 °C at 10 °C/min and then were cooled back to 10 °C at 3 °C/min. Note that the solid polymers were obtained by lyophilization of the corresponding micellar solutions.

### Dynamic Light Scattering (DLS) and Static Light Scattering (SLS) Analyses

DLS and SLS measurements were performed on a Wyatt DAWN HELEOS-II instrument with a laser wavelength of 658 nm. CMDX, MCDX and RCMDX block copolymers were individually dissolved in 60 mM NaCl D_2_O or H_2_O stock solution with various pH values at room temperature (21 °C). The solutions were heated up to 80 °C for 2 h and then slowly cooled back to room temperature (21 °C) overnight. The sample solutions were filtered by the PES syringe filters (pore size = 0.45 μm) into precleaned 8 mL scintillation vials before DLS measurements. Each sample solution was measured at 25 °C for 15 min with a collection interval of 5 s. The DLS exponential decay curve was fitted by the cumulant method to obtain the average size of the hydrodynamic radius (*R*_h_).^52^

### Critical Micelle Concentration (CMC) Determination

Critical micelle concentrations of corresponding polymers have been determined by SLS measurements of a series of CMDX, MCDX, and RCMDX micellar solutions with different concentrations (Figure S10).^53,54^ The CMDX, MCDX, and RCMDX block copolymers were individually dissolved in 60 mM NaCl H_2_O solution at room temperature (22 °C), and the polymer solutions were thermally annealed at 80 °C for 2 h and slowly cooled to room temperature overnight to obtain the micellar solutions.

### Transmission Electron Microscopy (TEM) Analyses

TEM analyses were conducted on a JEM-1400 TEM instrument operated at 80 kV in the Shared Instrument Facility at Louisiana State University (Baton Rouge, LA). Five μL portion of the 0.05 mg/mL CMDX, MCDX and RCMDX micellar solutions (pH ∼ 9) was applied to a carbon coated 300 mesh copper grid (Electron Microscopy Sciences) and the excess liquid was removed by filter papers to form a thin sample film. Then the grids were stained with uranyl acetate for 1 min.

### ζ-Potential Measurements

ζ-Potential measurements were conducted on a Malvern Zetasizer Nano ZS instrument using a laser wavelength of 633 nm at room temperature (22 °C). The polypeptoid BCP micellar solutions in D_2_O were injected into precleaned folded capillary cells for measurement. The applied voltage for the measurements is 50 V. Measurements are reported as the average of three measurements for each sample. The sample list and details of the ζ-potential can be found in Table S4.

### Small-Angle Neutron Scattering (SANS) Measurements

SANS measurements of the CMDX, MCDX and RCMDX micellar solutions were conducted at the Oak Ridge National Laboratory (ORNL; Oak Ridge, TN) on the Bio-SANS instrument, using neutron wavelength as λ = 6 Å and a wavelength spread of Δλ/λ = 13.2%. The temperature was maintained at 20 ± 0.1 °C using a Peltier temperature controller. The *q* range of the measurements spanned from ∼0.003 Å^–1^ to ∼0.85 Å^–1^, where the scattering vector *q* was calculated from *q* = 4π sin θ/λ and 2θ is the scattering angle. All samples were measured in banjo cells with a path length of 2 mm mounted on a temperature-controlled sample holder. SANS data reductions were performed using the facility-wide developed drtsans package,^55^ which consisted of instrument dark, pixel sensitivity and solid angle corrections, and normalization to sample transmission and thickness and subtraction of empty cell scattering. The final output as a single 1D SANS profile contained the data from the two detector arrays stitched together and placed on an absolute scale, *I*(*Q*) (cm^–1^). The background (incoherent and coherent background) subtractions were performed by subtracting constant scattering intensity values (∼0.55 cm^–1^) from the background via Igor Pro software. The aggregation number and radius of gyration of the micelles can be obtained from Guinier analysis (eqs S1 and S2).^56^ The results are summarized in Table S2.

### Small-Angle X-ray Scattering (SAXS) Measurements

SAXS measurements of CMDX and MCDX block copolymer micellar solutions were conducted at the Cornell High Energy Synchrotron Source (CHESS, Ithaca, NY) on the ID7A1 Bio-SAXS beamline SAXS instrument with an X-ray wavelength of λ = 1.25 Å (which can be calculated from the X-ray energy of 9.93 keV). Small-angle X-ray scattering (SAXS) data were collected on an EIGER 4 M detector. The temperature was maintained at 20 ± 0.1 °C using a circulating bath. The *q*-range of the measurements can be covered from ∼0.008 Å^–1^ to ∼0.6 Å^–1^, where *q* is the scattering vector that can be calculated from *q* = 4π sin θ/λ. The solvent and coherent background was subtracted by the buffer solution (60 mM NaCl aqueous solutions with different pHs) via RAW software. Sample solutions were loaded and measured in a quartz capillary (diameter = 1.5 mm, wall thickness = 0.01 mm) flow-cell at 20 °C. Fifty images for each CMDX and MCDX solution samples and background solutions (i.e., a buffer solution of H_2_O with 60 mM NaCl, pH = ca. 2 - 12) were measured. The SAXS measurements of RCMDX micellar solutions were conducted at the Advanced Photon Source (Argonne National Laboratory, Lemont, IL) on the 12-ID-B beamline SAXS instrument with an X-ray wavelength as λ = 0.886 Å (which can be calculated from the X-ray energy of 14.0 keV). Small-angle X-ray scattering data were collected using a Pilatus 2 M detector (DECTRIS Ltd.). The temperature was maintained at 20 ± 0.1 °C using a circulating bath. The *q* range (*q* = 4π sin θ/λ) of the measurements can be covered from ∼0.003 Å^–1^ to ∼0.9 Å^–1^, where θ is the Bragg angle. Samples were measured by using a quartz capillary flow cell at 20 °C. The diameter of the capillary is 1.5 mm, and the wall thickness is 0.01 mm. Twenty images for each RCMDX solution sample and background solution (i.e., a buffer solution of H_2_O with 60 mM NaCl, pH = ca. 2 - 12) were measured. The 2D images were converted to 1D SAXS data and averaged using matSAXS package provided by beamline 12-ID-B (https://12idb.xray.aps.anl.gov/Software_Processing.html). The SAXS signals of the buffer background were subtracted using RAW software^57^ and SasView software (http://www.sasview.org/) was used for further data analysis. The SAXS data were best fitted with core–shell ellipsoidal model (eqs S3–S6), and the results were summarized in Table S3.

### p*K*_a_ Determination

The p*K*_a_ values of the CMDX, MCDX, and RCMDX micellar solutions were determined by titrating 5.0 mg/mL solution samples with 0.25 M NaOH or 0.25 M HCl solution at 21 °C. The p*K*_a_ values were obtained by the maximum second-order derivative of the titration curves. The detailed procedures are listed below. The polymers were dissolved into ultrapure water with 60 mM NaCl, and the polymer solutions were thermally annealed at 80 °C for 2 h and cooled to room temperature (22 °C) overnight before titration. NaOH stock solution (0.25 M) was added gradually into the aqueous solution of CMDX, MCDX, and RCMDX (5.0 mg/mL) with stirring, respectively. The pH values were plotted against the volume of the NaOH addition. The p*K*_a,1_ value, which corresponds to the ionization of carboxyl groups(CO_2_H) in the respective micelles can be calculated by using the Henderson–Hasselbach equation. Similarly, the p*K*_a,2_ value corresponding to the ionization of secondary amine groups (NR_2_H) in the respective micelles can be obtained by titrating the micellar solutions with HCl solution (0.25 M).

## Results and Discussion

### Synthesis and Characterization of the Ionic Polypeptoid BCPs

To investigate the effect of charge pattern on the micellar structure in aqueous solution, three amphoteric polypeptoid block copolymers (BCPs) with a head–tail asymmetry and two ionizable sites along the chain were designed and synthesized (Scheme 1). One ionizable site is fixed at the hydrophobic terminus of the chain, while the other ionizable site is positioned either at the hydrophilic segment terminus, at the hydrophilic-and-hydrophobic junction, or randomly distributed along the hydrophilic segment (Scheme 1). The polypeptoid BCPs are comprised of three different *N*-substituted glycine monomeric units, namely, *N*-2-carboxyethyl glycine (C), *N*-2-methoxyethyl glycine (M), and *N*-decyl glycine (D), with a targeted number-average degree of polymerization (DP_n_) = 25. The targeted hydrophilic segments are composed of 19 *N*-2-methoxyethyl glycine (M) and one *N*-2-carboxyethyl glycine (C) monomeric units which can be ionized to carry a negative charge. The targeted hydrophobic segments are composed solely of five *N*-decyl glycine (D) monomeric units including the terminal unit carrying the ionizable secondary amine functionality (X).

These three polypeptoid BCPs, namely poly(*N*-2-carboxyethyl glycine)-*b*-poly(*N*-2-methoxyethyl glycine)-*b*-poly(*N*-decyl glycine) (CMDX), poly(*N*-2-methoxyethyl glycine)-*b*-poly(*N*-2-carboxyethyl glycine)-*b*-poly(*N*-decyl glycine) (MCDX), and poly(*N*-2-carboxyethyl glycine-*r*-*N*-2-methoxyethyl glycine)-*b*-poly(*N*-decyl glycine) (RCMDX) were synthesized by the *N*,*N*-dimethylamine-initiated sequential ring-opening polymerization (ROP) of the corresponding *N*-substituted glycine derived *N*-carboxyanhydride monomers (i.e., MeOEt-NCA, ^t^BuCO_2_Et-NCA, and De-NCA) in 50 °C THF followed by TFA treatment to uncloak the carboxylic acid functionality on the side chain (Scheme 1).^43,50,51^ Each step of the polymerization reached quantitative conversion prior to the sequential addition of new monomers to produce block copolymers. The polymers were purified by dialysis and dried by lyophilization to a white, fluffy powder prior to further characterization. The synthetic details are provided in the Supporting Information.

The block copolymer composition (Table 1) was determined by ^1^H NMR analysis (Figure S3, S5, and S7) using the integration of *N*,*N*-dimethyl end-group proton signals at 2.95 ppm relative to that of the characteristic methyl protons of the *N*-decyl glycine unit at 0.87 ppm, the methylene protons of the *N*-2-carboxyethyl glycine unit at 2.56 ppm, and the methylene protons on the backbone of all monomeric units in the 3.75–4.50 ppm range. Considering the uncertainty of the integration values in ^1^H NMR analysis, CMDX and MCDX polymers are considered to have identical compositions that agree well with the initial monomers-to-initiator ratio. The RCMDX polymer exhibited a slightly larger ionizable C monomer content (8.2 vol %) and lower hydrophobic D monomer content (32 vol %) relative to the CMDX and MCDX polymers (∼5 vol % and ∼36 vol % for C and D monomers, respectively, Table S1). The absolute polymer molecular weight (*M*_n_) and polydispersity index (*Đ* = *M*_w_/*M*_n_; Table 1) were determined by size-exclusion chromatography with tandem differential refractive index and multiangle light scattering technique (SEC-dRI-MALS) in 1,1,1,3,3,3-hexafluoro-2-propanol (HFIP) with 3.0 mg/mL CF_3_CO_2_K. The RCMDX polymer exhibited a monomodal peak with a narrow molecular weight distribution (*Đ* = 1.03), whereas CMDX and MCDX polymers exhibited a major peak with a minor shoulder at shorter elution time in their respective SEC-dRI chromatograms (Figure S8). The minor peaks were attributed to the presence of polymer aggregates in the HFIP/CF_3_CO_2_K solvent, resulting in broader molecular weight distribution for CMDX (*Đ* = 1.21) and MCDX polymers (*Đ* = 1.22), relative to that of RCMDX (Table 1), respectively. Analysis of the major peak in the SEC chromatograms alone afforded reduced *Đ* values of 1.03 and 1.07 for the CMDX and MCDX polymers, respectively. These combined characterization results confirm the successful synthesis of the targeted polypeptoid block copolymers.

**Table 1:** Molecular Characteristics of Polypeptoid BCPs (CMDX, MCDX, RCMDX)

[M_C_]_0_:[M_M_]_0_:[M_D_]_0_:[I]_0_^a^	polymer composition^b^	*M*_n_ (theo.) (g/mol)^c^	*M*_n_ (NMR) (g/mol)	*M*_n_ (SEC) (g/mol)^d^	*Đ* (SEC)^d^
1:19:5:1	C_1.2_M_17_D_4.7_X	3350	3300	3600 (4100)	1.21 (1.03)
1:19:5:1	M_18_C_1.1_D_4.7_X	3350	3400	3300 (3600)	1.22 (1.07)
1:19:5:1	RC_1.9_M_18_D_3.9_X	3350	3400	2700	1.03

a The initial monomer-to-initiator ratio in the polymerization.

b The subscript signifies the number-average degree of polymerization (DP_n_) of *N*-2-carboxyethyl glycine (C), *N*-2-methoxyethyl glycine (M), and *N*-decyl glycine unit (D), respectively, as determined by ^1^H NMR spectroscopy. X represents the terminal hydrophobic monomer unit bearing the ionizable secondary amine end-group; R signifies the random distribution C and M monomers in the hydrophilic segment of the designated BCP.

c The theoretical molecular weights were calculated based on the initial monomer-to-initiator ratio. Note that each step of the polymerization reached quantitative conversion.

d The experimental molecular weights and polydispersity indexes were determined by SEC-dRI-MALS method in HFIP/CF_3_CO_2_K (3.0 mg/mL) at 40 °C (d*n*/d*c* = 0.230 mL/g). The *M*_n_ and *Đ* values in the brackets were obtained by analyzing the major peak in SEC chromatograms and excluding the minor shoulder peak due to polymer aggregation.

### Determination of CMC, p*K*_a_, and pI of Ionic Polypeptoid BCP Micelles

Ionic polypeptoid BCPs, namely, CMDX, MCDX, and RCMDX, were separately dissolved in deionized water with a constant 60 mM NaCl concentration and varying polymer concentrations between 0.002 and 5.0 mg/mL. All solutions were thermally annealed at 80 °C for 2 h to ensure complete polymer dissolution and equilibration before standing at room temperature (21 ± 2 °C) overnight to attain the final micellar solutions. Note that DSC analysis of the lyophilized CMDX, MCDX, and RCMDX micellar solutions revealed a broad endotherm centered at 76, 72, and 62 °C in the first heating cycle, respectively (Figure S9), suggesting that thermal annealing of the micellar solutions at 80 °C facilitates equilibration of polymer micelles in solution.

Static light-scattering (SLS) measurements of the micellar solutions were conducted to determine the critical micelle concentration (CMC; Figure 2a).^53,54^ The CMDX, MCDX, and RCMDX polymers were found to form micelles with comparable CMC at 0.17, 0.15, and 0.18 mg/mL in an aqueous solution with 60 mM NaCl concentration at 25.0 °C, respectively (Table 2).

**Figure 1:**
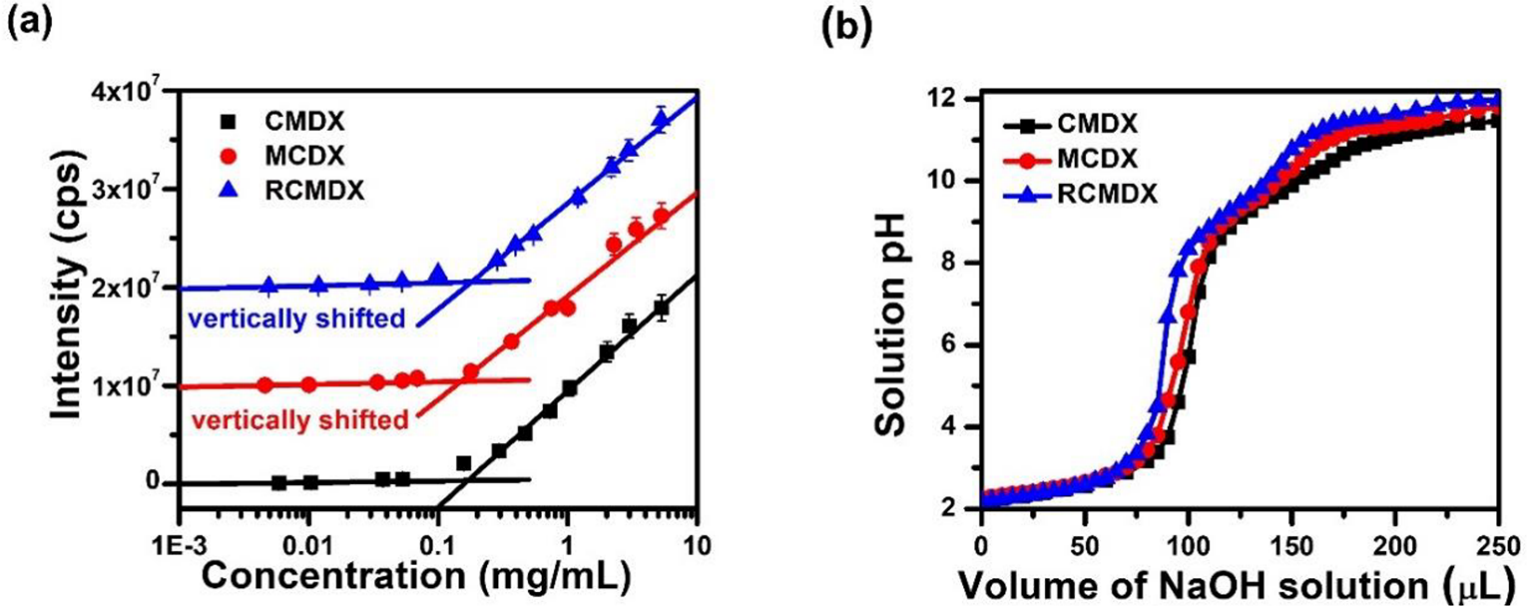
(a) Plots of light scattering intensity vs polymer concentration to determine CMC of CMDX, MCDX, and RCMDX micelles in aqueous solution ([NaCl] = 60 mM, 25 °C). MCDX and RCMDX data and fitting curves were vertically shifted by a factor of 5 × 10^6^ and 1 × 10^7^ for clarity. (b) Titration curves of CMDX, MCDX, and RCMDX micellar solution (polymer concentration = 5.0 mg/mL, [NaCl] = 60 mM, 21 ± 2 °C).

**Table 2:** Critical Micelle Concentration (CMC), p*K*_a_, and pI of Polypeptoid BCP Micelles^a^

polymer abbreviation	CMC (mg/mL)	p*K*_a,1_	p*K*_a,2_	pI^b^
CMDX	0.17	2.5 ± 0.1	9.5 ± 0.3	6.0 ± 0.2
MCDX	0.15	2.5 ± 0.3	9.9 ± 0.1	6.2 ± 0.2
RCMDX	0.18	2.4 ± 0.1	9.7 ± 0.4	6.1 ± 0.2

a CMC, p*K*_a_ and pI were determined for the polypeptoid BCP micellar solution in water at 5.0 mg/mL polymer concentration and 60 mM NaCl concentration at 25 °C (CMC) or 21 ± 2 °C (p*K*_a_ and pI).

b pI values were determined by pI = (p*K*_a,1_ + p*K*_a,2_)/2.

**Figure 2:**
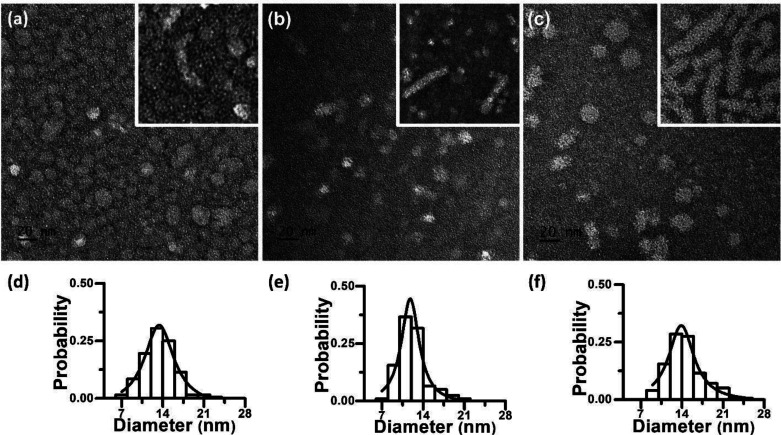
Representative TEM images and the micellar diameter distribution profiles (*n* = 200) of spherical micelles of CMDX (a, d), MCDX (b, e), and RCMDX (c, f) polymers, respectively. The sporadic presence of short rod-like micelles in the micellar samples were also displayed in their respective inset. The scale bar in (a)–(c) is 20 nm. The micellar samples were stained with uranyl acetate prior to TEM imaging. The histograms were fitted with Lorentz function to determine the mean micellar diameter.

TEM analysis of the CMDX, MCDX, and RCMDX micellar solution at pH = 9 revealed mostly micelles with spherical geometry and comparable size (Figure 2). The mean diameters of CMDX, MCDX and RCMDX spherical micelles were found to be 13 ± 2, 12 ± 3, and 14 ± 3 nm based on the measurements of a total number (*n*) of 200 particles in the transmission electron micrographs, respectively. Occasionally, short rod-like micelles were also observed in the TEM images of CMDX, MCDX, and RCMDX (Figure 2, insets, and Figure S11). The width of the rod-like micelles was found to be 16 ± 3 nm (CMDX), 15 ± 3 nm (MCDX), and 13 ± 2 nm (RCMDX) (*n* = 30), which are comparable to the diameter of the more abundant spherical micelles observed in all samples. The rod-like micelles were relatively short with the contour length varying from 36 ± 9 to 57 ± 23 to 38 ± 10 nm and aspect ratio varying from 2.3 ± 0.6 to 3.9 ± 1.7 to 3.0 ± 0.7 for the CMDX, MCDX, and RCMDX samples, respectively.

To determine the p*K*_a_ and ionization state of the micelles at different solution pHs, titration studies were conducted for the CMDX, MCDX and RCMDX micellar solutions at 5.0 mg/mL with 60 mM NaCl and at 22 °C.^5^ The titration curve revealed the presence of two ionizable sites for CMDX, MCDX and RCMDX micelles corresponding to the *N*-2-carboxyethyl glycine unit and the *N*-decyl glycine chain terminus bearing a secondary amine group. The corresponding p*K*_a,1_ was found to be 2.5 ± 0.1, 2.5 ± 0.3, and 2.4 ± 0.1; the p*K*_a,2_ was 9.5 ± 0.3, 9.9 ± 0.1, and 9.7 ± 0.4 for the CMDX, MCDX, and RCMDX micelles, respectively. The isoelectric point (IEP) determined from pI = (p*K*_a,1_ + p*K*_a,2_)/2 was found to be 6.0 ± 0.2, 6.2 ± 0.2, and 6.1 ± 0.2 for CMDX, MCDX, and RCMDX micelles, respectively (Figure 1b, Table 2). This result indicates that the thermodynamic propensity for ionization is nearly identical for these three micelle types and the state of ionization of the micelles can be effectively controlled by the solution pHs.

### Small-Angle Neutron Scattering (SANS) Analysis of Polypeptoid BCP Micellar Solutions

To investigate the effect of the charge pattern on the polypeptoid BCP micellar structure in an aqueous solution, a series of MCDX, CMDX, and RCMDX solutions in D_2_O or H_2_O with varying pHs in the 2–12 range and a constant 5.0 mg/mL polymer concentration and 60 mM NaCl concentration were prepared and thermally annealed at 80 °C for 2 h, followed by cooling to room temperature overnight prior to SANS or SAXS measurements at 20 °C. Note that pH values of all micellar solutions in D_2_O were obtained by using the following relation: pH = 0.929 × pH* + 0.42, where pH* is the direct reading of a H_2_O-calibrated pH meter in a D_2_O solution.^58^

**Figure 3:**
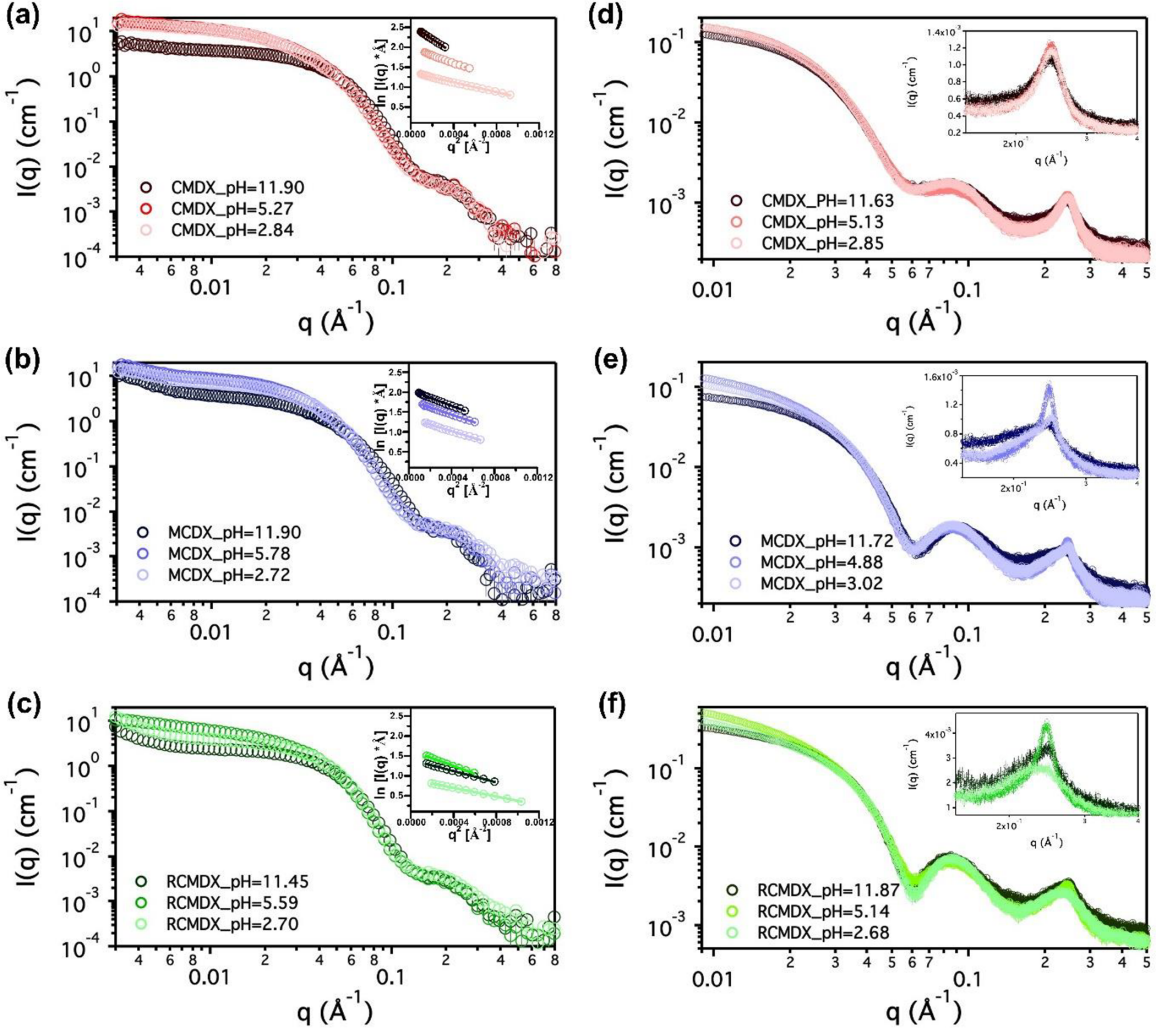
(a–c) SANS intensity profiles and Guinier analysis (inset) for (a) CMDX, (b) MCDX, and (c) RCMDX at three selected pHs (ca. 2, 5, and 12); (d–f) SAXS intensity profiles and high *q* diffraction peak (inset) of (d) CMDX, (e) MCDX, and (f) RCMDX at three selected pHs (ca. 2, 5, and 12). All scattering experiments were conducted using [polymer] = 5.0 mg/mL and [NaCl] = 60 mM in D_2_O (SANS) or H_2_O (SAXS) solvents at 20 °C.

Figure 3a–c displays representative SANS profiles of CMDX, MCDX, and RCMDX micellar solutions at three selected pHs spanning the 2–12 range. SANS profiles at additional pH values are shown in Figure S12 in the Supporting Information. In the low *q* region (0.007 < *q* < 0.015 Å^–1^), the scattering intensities of all SANS data exhibited a power-law dependence on *q* (i.e., *I* ∼ *q*^α^) with an exponent (α) of −0.2 ∼ −0.4, −0.3 ∼ −0.4, −0.2 ∼ −0.5 for CMDX, MCDX, and RCMDX micelles, respectively, indicating the formation of a zero-dimensional nanostructure in these polymer solutions, in agreement with the TEM analysis (Figure 2). In addition, in the low-to-middle *q* region (0.01 < *q* < 0.03 Å^–1^), the scattering intensities were found to first increase with increasing pH from ∼2 to ∼6 and then decrease with a further pH increase between ∼6 and ∼12 for the MCDX and RCMDX micelles. By contrast, in the case of CMDX micelles, the scattering intensities did not change significantly in the ∼2–6 pH range and then steadily decreased with increasing pH above ∼6. These combined results indicate that the changes in solution pH have induced structural reorganization of the micelles. Furthermore, the SANS scattering intensity profiles in the very low *q* region (0.003 < *q* < 0.008 Å^–1^) gradually developed a discernible upturn with increasing pH for the MCDX and RCMDX micelles, indicating the presence of larger aggregates in these micellar solutions in low abundance, consistent with the TEM observation (Figure S11). Finally, it should also be noted that a weak and broad peak centered at *q* ≈ 0.23 Å^–1^ was discernible in the high *q* range (0.1 < *q* < 0.3 Å^–1^) for all three micelles, the origin of which will be discussed later.

**Figure 4:**
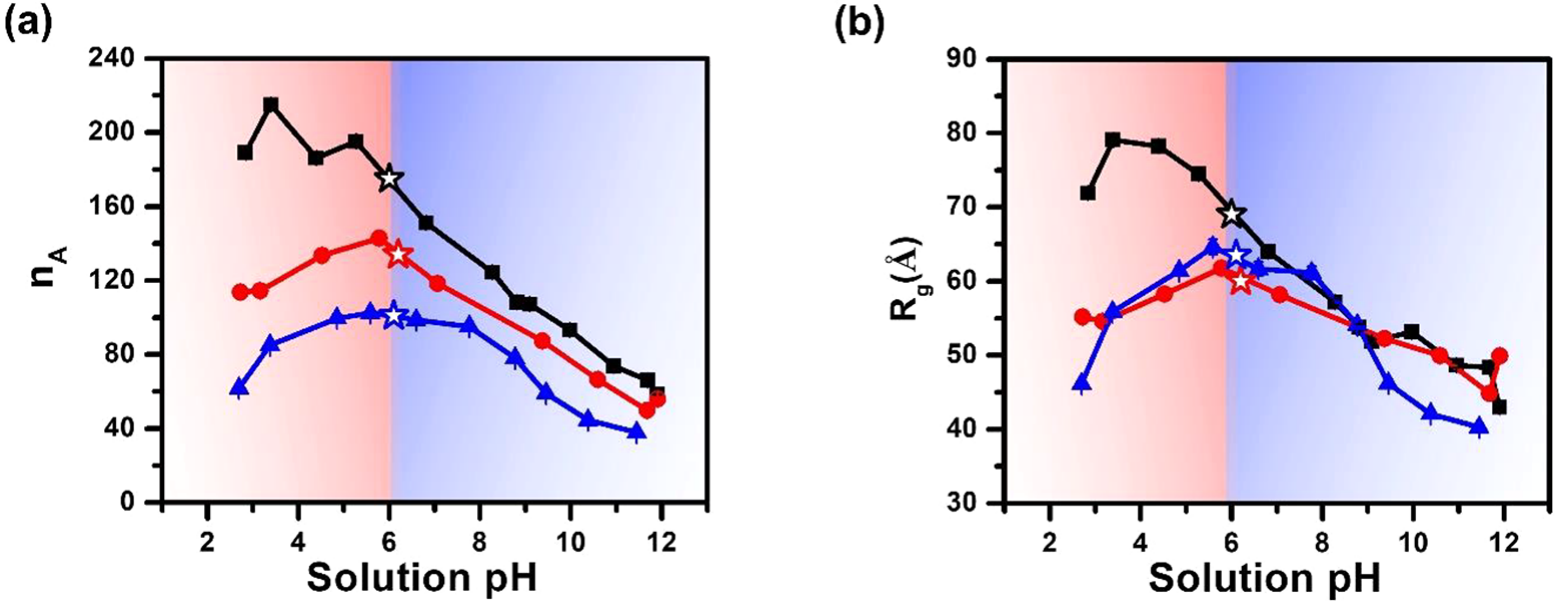
(a) Plot of aggregation number (*n*_A_) and (b) radius of gyration (*R*_g_) of CMDX (black square and line), MCDX (red circle and line), and RCMDX (blue triangle and line) micelles vs solution pHs. The isoelectric point (IEP) corresponding to each micelle type is indicated by an opened star symbol.

Guinier plot analysis was performed on the SANS data collected at different pHs in the low *q* region (0.01 < *q* < 0.02 Å^–1^) with the criterium of (0.5 < *R*_g_·*Q*_max_ ≤ 1.3) to determine the radius of gyration (*R*_g_) and aggregation number (*n*_A_) of the micelles in a model-independent manner (Figure 3).^55^ All three micelle types exhibited varying levels of structural reorganization as the solution pH is altered, evidenced by the notable change of *n*_A_ and *R*_g_ as a function of solution pH (Figure 4). Interestingly, the dependence of *n*_A_ on the solution pH was found to parallel that of *R*_g_ for all micelles. There is a discernible presence of two regimes where dependence of the micellar structure on the solution pH differs, and the boundary of two regimes meets approximately at the isoelectric point (IEP). The IEP is indicated by an unfilled star on each profile. At pHs below the IEP (pH < 6), the CMDX micelles exhibited high values of *n*_A_ = 190–210 and *R*_g_ = 72–79 Å, which then steadily decreased to *n*_A_ = 60 and *R*_g_ = 43 Å (pH = 12) with increasing solution pH above the IEP. By contrast, the MCDX micelles exhibited a steady increase from *n*_A_ = 114 and *R*_g_ = 55 Å at pH = 2 to a maximum of *n*_A_ = 143 and *R*_g_ = 62 Å at approximately the IEP (pI = ∼ 6). With further increase of pH above the IEP, a decline was observed until *n*_A_ = 56 and *R*_g_ = 50 Å at pH = 12. Interestingly, RCMDX micelles exhibited a similar dome-shaped dependence of *n*_A_ and *R*_g_ to solution pH, as observed for MCDX micelles, except the decline is more gradual for the latter. It is also noteworthy that *n*_A_ of CMDX is largest for all given solution pH, followed by MCDX and RCMDX. On the other hand, the sample with the largest *R*_g_ of the three micelle systems varies with the solution pH, evidenced by the presence of multiple crossover points in the *R*_g_ versus pH plot. This indicates a notable difference in the chain packing in these three micelle types at any given solution pH. Another intriguing point is that the MCDX and CMDX exhibited a much more pronounced difference in *n*_A_ and *R*_g_ at any given pH below IEP than above it, indicating that the position of the ionizable *N*-2-carboxyethyl glycine monomer along the polymer chain influences the micellar structure in a pH-dependent manner (vide infra).

### Small-Angle X-ray Scattering (SAXS) Analysis of Polypeptoid BCP Micellar Solutions

SAXS measurements of CMDX, MCDX, and RCMDX micellar solution (5.0 mg/mL polymer concentration, [NaCl] = 60 mM) in H_2_O at different pHs were conducted with a flow cell setup at 20 °C (Figure 3d–f). SAXS intensity profiles of all three micelles exhibited a power-law dependence on *q* (i.e., *I* ∼ *q*^α^) with an exponent (α) of −0.5 ∼ −0.7, −0.4 ∼ −0.8, and −0.3 ∼ −0.8, for CMDX, MCDX, and RCMDX, respectively, in the low *q* region (0.009 < *q* < 0.012 Å^–1,^), which are slightly higher than those of SANS profiles. In the mid *q* range, SAXS profiles exhibited a notable minimum at *q* = 0.05 Å^–1^, consistent with the formation of a core–shell type micelles. This feature is distinctly absent in the SANS profiles due to the reduced neutron scattering length density (SLD) contrast between the micellar core and shell, resulting from solvent penetration into the micelles (Table S2). Consistently, the changes of slope (or the knee feature) past the Guinier region occurred at a much lower *q* range for all micellar solution samples in their SAXS profiles relative to those in the corresponding SANS profiles, suggesting larger apparent micellar sizes observed in SAXS than SANS measurements. This is attributed to the reduced neutron SLD contrast between the micelles and bulk solvent in the SANS measurements relative to the X-ray SLD contrast in SAXS measurements due to solvent penetration. In the high *q* region, all micellar solutions exhibited a pronounced peak at *q* ≈ 0.23 Å^–1^ in the SAXS profiles, which is significantly attenuated in the SANS profiles (Figure 3).

As the SAXS profiles of all micelles exhibited more pronounced spectral features consistent with a core–shell structure relative to the SANS profiles, we conducted model fitting of SAXS profiles to obtain more detailed structural information on these solution micelles. All SAXS profiles can be best-fitted using a core–shell ellipsoidal model (eqs S1–S4).^59,60^ The sphere, core–shell sphere, or ellipsoidal models failed to adequately fit the SAXS data. The details of the model fitting together with the representative fitting curves (Figure S13) and micellar structural parameters obtained from model fitting (Table S3) are provided in the Supporting Information.

**Figure 5:**
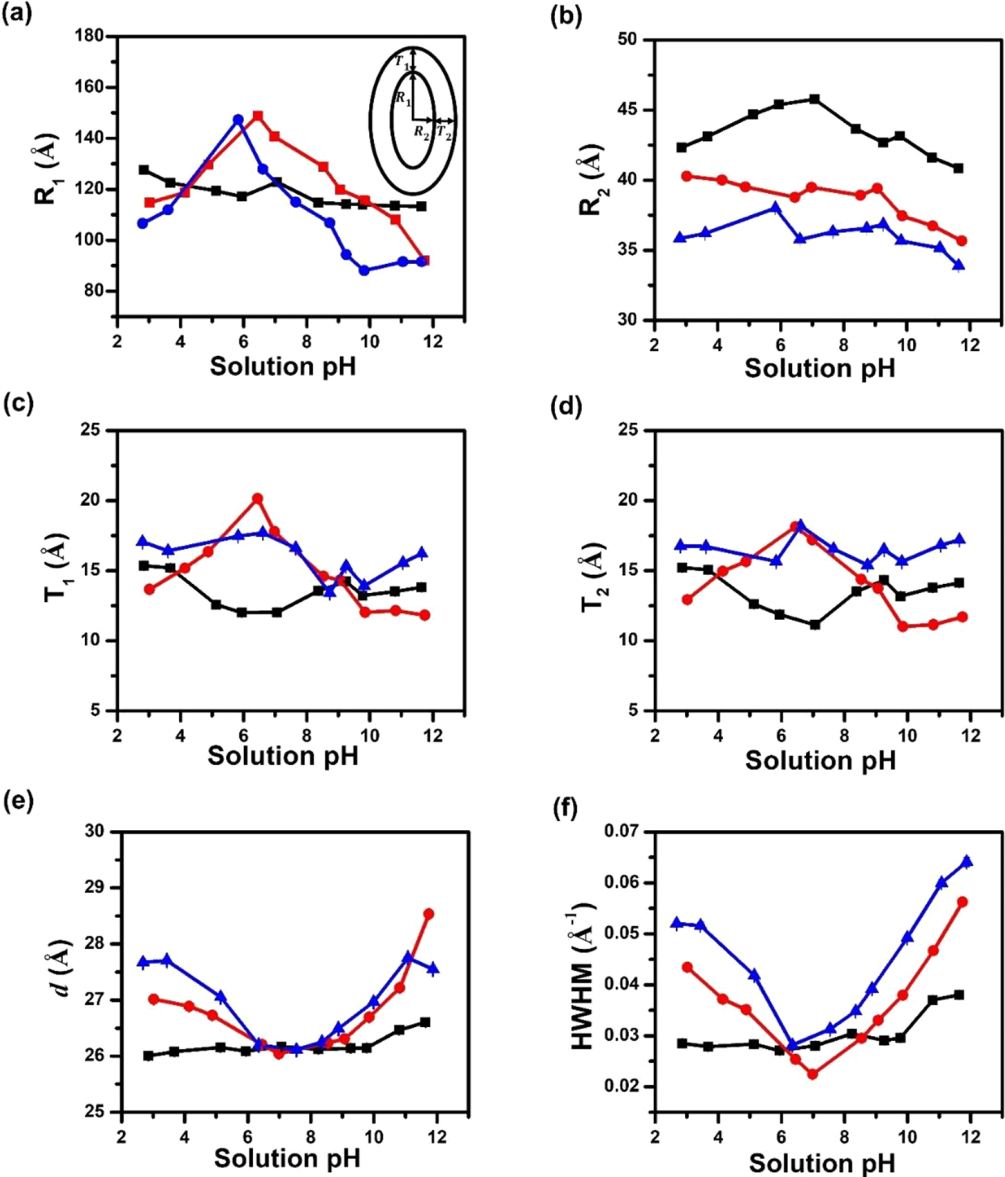
Structural parameters of the CMDX (black square and line), MCDX (red circle and line), and RCMDX micelles (blue triangle and line) at different solution pHs obtained by best-fits of their respective SAXS profiles using the core–shell ellipsoidal model. (a, b) Plots of the micellar core radius along the long (*R*_1_) and short axis (*R*_2_), (c, d) micellar shell thickness along the long (*T*_1_) and short axis (*T*_2_), and (e, f) the characteristic *d*-spacing and half-width at half-maximum (HWHM) of the SAXS scattering peak at q* = 0.23 Å^–1^ versus solution pH for the CMDX, MCDX, and RCMDX micelles, respectively.

The structural parameters obtained from the model fitting provide an estimation of the core radius and shell thickness along the long and short axis of the ellipsoidal micelles. It was found that the core radius along the long axis of the ellipsoidal micelles (*R*_1_) is approximately three to four times that along the short axis (*R*_2_), whereas the shell thickness along both long and short axis (*T*_1_ and *T*_2_) is similar for all micelles at any given solution pH. This suggests that these micelles shared a common uniform corona thickness and a nonspherical core. For the CMDX micelles, the core radius along the long axis of the micelles (*R*_1_) was found to decrease slightly from 128 to 113 Å with increasing pH from ∼2 and ∼12 (Figure 5a). By contrast, the MCDX and RCMDX micelles exhibited a stronger and dome-shaped dependence of *R*_1_ on the solution pH with a maximum *R*_1_ of ∼150 Å at about IEP (pI ∼ 6). Departure of the solution pH from the IEP is correlated to a steady decline of the *R*_1_ to ∼110 Å at pH ∼ 2 and to ∼90 Å at pH ∼ 10 or ∼12 for the MCDX and RCMDX micelles, respectively (Figure 5a). The core radius along the short axis (*R*_2_) did not change significantly with the solution pH for all three micelle types; the CMDX micelles have the largest mean value of *R*_2_ = 43 ± 3 Å followed by MCDX (*R*_2_ = 39 ± 3 Å) and then RCMDX (*R*_2_ = 36 ± 2 Å; Figure 5b). By comparison, the corona dimensions of three micelle types are comparable along both long and short axis of the micelles, evidenced by the mean value of the shell thickness of 14 ± 2, 14 ± 4, and 17 ± 1 Å for the respective CMDX, MCDX, and RCMX micelles in the entire 2–12 pH range (Figure 5c-5d).

In the high *q* region, all micellar solutions exhibited a pronounced peak at q* ≈ 0.23 Å^–1^ in the SAXS profiles, which is significantly attenuated in the SANS profiles (Figure 3). This peak is consistent with the characteristic dimension along the crystallographic *c*-axis (*d* = 25 Å) of a sanidic liquid crystalline phase in the micellar core where the poly(*N*-decyl glycine) (D) segments adopt a nearly planar molecular geometry and stack into a lamellar arrangement.^61−63^ The formation of the sanidic liquid crystalline phase has previously been observed by SAXS measurements of the 1D and 2D solution micelles of nonionic polypeptoid BCPs containing the solvophobic D segments.^64,65^ The weak diffraction peak in the SANS profile is due to the poorly monochromatic neutron beam (Δλ/λ = 0.132) and strong incoherent scattering from the large number of hydrogen atoms in the sample. Note that a constant incoherent background intensity of ∼0.55 cm^–1^ was subtracted from the SANS data.

The characteristic peak was fitted using a Lorentz function to determine the peak position and half-width at half-maximum (HWHM) and plotted as a function of the solution pH (Figure 5e,f). For the CMDX micelles, the characteristic *d*-spacing determined using *d* = 2π/q* was found to remain nearly constant at 26.1 ± 0.1 Å between pH 2 and 10 and increase slightly to 26.6 Å with an increase in solution pH to 12 (Figure 5e). By contrast, the *d*-spacing of the MCDX and RCMDX micelles follows an inverse dome-shaped dependence to solution pH, with a minimum observed at ∼26.0 Å at pH 7. Further, the *d*-spacing steadily increased to ∼27.0–28.5 Å as the solution pH either decreased to ∼2 or increased to ∼12. These results indicated that the chain packing progressively transitions to a less compact conformation in the micellar core of MCDX and RCMDX micelles as the solution pH is either increased or decreased from ∼ pH 7. In contrast, the chain packing in the CMDX micellar core is more compact and much less perturbed by the change in the solution pH.

The HWHM is indicative of the long-range correlation of the molecular ordering in the liquid crystalline phase.^66^ Dependence of the HWHM on the solution pH was found to follow a similar trend as that of the characteristic *d*-spacing for the respective CMDX, MCDX, and RCMDX micelles (Figure 5f). The HWHM for CMDX micelles remained nearly constant at 0.028 ± 0.001 Å^–1^ between pH 2 and pH 10 and increased moderately to 0.038 Å^–1^ with a further increase of pH to 12, indicating that long-range molecular ordering of D segments in the CMDX micellar core is not significantly perturbed by solution pH. By contrast, both MCDX and RCMDX micelles exhibited an inverse dome-shaped dependence of HWHM to solution pH. The minimum in the HWHM was observed at 0.022 Å^–1^ (pH ∼ 7) for MCDX and 0.028 Å^–1^ (pH ∼ 6) for RCMDX. The HWHM value steadily increased to 0.056 Å^–1^ for MCDX and 0.064 Å^–1^ for RCMDX at pH ∼ 12 and to 0.043 Å^–1^ for MCDX and 0.052 Å^–1^ for RCMDX at pH ∼ 2. This is consistent with more long-range molecular ordering within the micellar core at pH ∼ 6–7 than when the pH is not in the neutral range. In addition, the HWHM values are comparable for CMDX, MCDX, and RCMDX samples for pH = 6–7. Departure from neutral pH resulted in progressively smaller HWHM for the CMDX micelles relative to that for the MCDX and RCMDX micelles, which indicated a more long-range molecular ordering in the micellar core for CMDX micelles.

It is evident that changing the ionization state of the *N*-2-carboxyethyl glycine (C) monomer positioned at the hydrophilic segment terminus has a much limited impact on the molecular packing of the solvophobic segment (D) in the CMDX micellar core. In contrast, for the MCDX and RCMDX micelles where all or some ionizable C monomers are located at the hydrophobic-and-hydrophilic segment junctions, the change of ionization state of these monomers significantly influences the molecular packing in their respective micellar core. We tentatively attribute this to strong solvation propensity of the ionizable C monomers, which can distort the chain conformation and packing in the micelles. Previously performed molecular dynamics (MD) simulations have revealed that positioning a charged monomer at the core–shell interface of micelles can cause significant distortion to the micellar shape due to strong propensity of the charged monomer to reside on the micellar surface and be maximally solvated and that micelles can also minimize the electrostatic repulsion by increasing counterion association when the micellar charge density is high.^11^ In addition, when the micelles carry nearly equal amounts of positive and negative structural charges near IEP (pI ∼ 6), they exhibited the most compact and long-range ordered molecular packing in the micellar core. The MCDX and RCMDX micelles followed this behavior. As the pH deviates from IEP, the micelles become increasingly charged and solvated; increasing counterion association with the net charges in the micelles leads to less compact micelles with reduced long-range ordered molecular packing in the micellar core.

### DLS Analysis of Polypeptoid BCP Micellar Solutions

**Figure 6:**
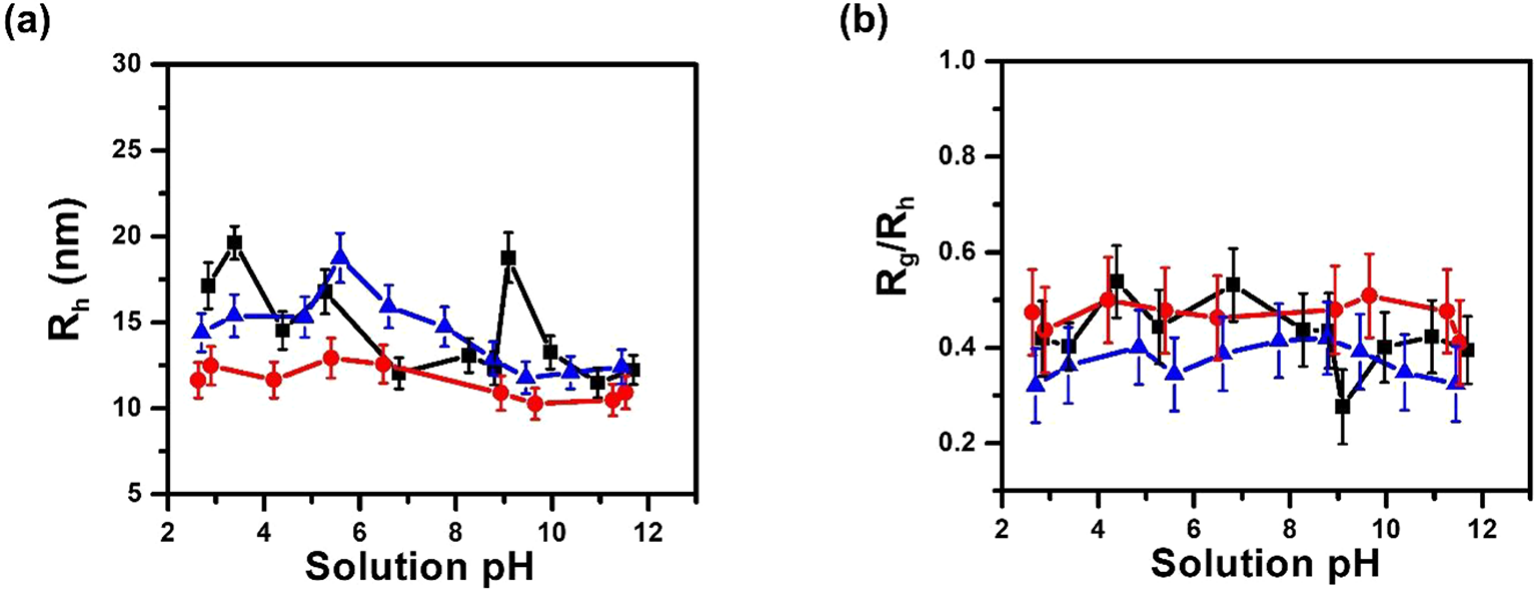
(a) Plots of hydrodynamic radius (*R*_h_) and (b) shape factor (*R*_g_/*R*_h_) vs solution pHs for CMDX (black square and line), MCDX (red circle and line), and RCMDX micelles (blue triangle and line) in dilute aqueous solution, respectively.

DLS analysis of polypeptoid BCP micellar solutions at different solution pH revealed the formation of particles with hydrodynamic radius (*R*_h_) in the 10–13 nm range for the MCDX micelles, 12–18 nm for the CMDX micelles, and RCMDX micelles in the 2–12 pH range. Given the error bar in the *R*_h_ value, the hydrodynamic size of all three types of micelles does not change significantly with solution pH in the entire 2–12 pH range (Figure 6a). The shape factors (*R*_g_/*R*_h_) of all three types of micelles are in the 0.28–0.54 range (Figure 6b), consistent with a highly solvated core–shell micellar structure.^67,68^

### ζ-Potential Measurements of Ionic Polypeptoid BCP Micellar Solutions

**Figure 7:**
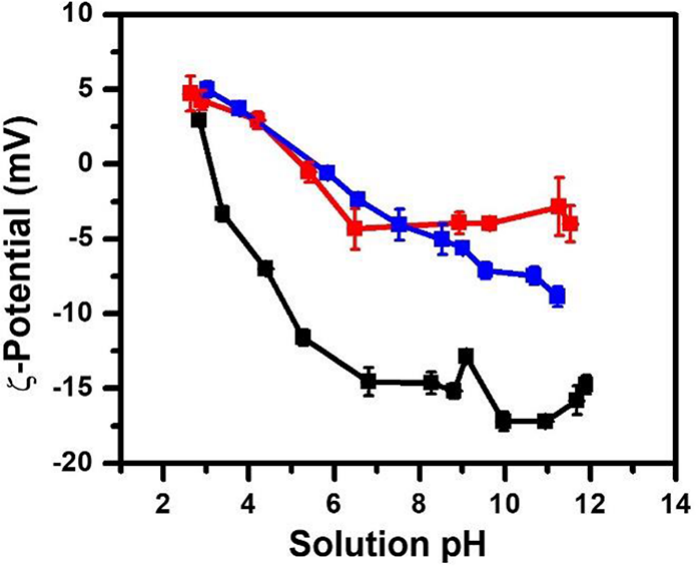
Plot of ζ-potential of CMDX (black square and line), MCDX (red square and line), and RCMDX (blue square and line) polypeptoid BCP micelles vs the solution pH values.

ζ-potential of CMDX, MCDX and RMCDX micelles were measured by electrophoretic methods at different solution pHs.^69^ Three micelles exhibited notable differences in the dependence of their respective ζ-potential on the solution pH (Figure 7). The CMDX micelles exhibited a slightly positive ζ-potential of 3 mV at low pH (= 3), which decreased and plateaued at *ca.* – 15 mV between pH 6 and 12. The MCDX micelles follow a similar trend as the CMDX micelles except that the ζ-potential decreased more gradually and plateaued at a less negative ζ-potential of ca. −4 mV. In contrast, RCMDX micelles exhibited a nearly linear decrease of the ζ-potential from +5 to −9 mV with increasing pH ∼ 2 to 12. Another point to note is that the ζ-potential for CMDX and MCDX micelles plateaued in the pH range 6–7, which also coincides with the IEP (pI ∼ 6).

These results indicate that the position of the ionizable monomers along the chain effectively modulate the ζ-potential of the amphoteric polymer micelles in solution. At the lowest solution pH ∼ 2 (<p*K*_a,1_), the polymers in the micelle bear the most positive charges and the ζ-potential of the three micelle types converged to a value of ∼5 mV. The small positive value of ζ-potential suggests that positive charges (NR_2_H_2_^+^) are located at the micellar core–corona interface far from the micelle-bulk water interface.^70^ This scenario is likely considering the facially amphiphilic characteristic of the solvophobic segment (D) thus allowing water penetration into the micellar interior and charge distribution on the micellar core–corona interface. A previous study on the solution micellar structure of the polyisoprene–polystyrene block copolymer (PI-PS) bearing a charge end-group at the PI terminus has revealed that the charge groups are preferentially distributed at the micellar core–corona interface instead of the core interior when micelles were formed in polystyrene selective solvent.^34−37^

As the pH increases toward IEP, the net positive charge of the polymers in the micelles decreases due to increasing negative charge (CO_2_^–^) content of the polymers. Accordingly, the ζ-potential gradually decreases into the negative regime for all samples and with the CMDX micelles exhibiting the sharpest decline and the most negative ζ-potential. This result is consistent with the negative charged CO_2_^–^ groups of the polymers being located closest to the micellar surface in the CMDX micelles.^70^ At the IEP where micelles have zero net charge due to an equal number of positive and negative charges bound to the polymers, all micelles exhibited negative ζ-potentials, indicating that negative charge groups (CO_2_^–^) are located closer to the micellar surface, and the positive charge (NR_2_H_2_^+^) and negative charge groups (CO_2_^–^) are not in close proximity within the micelles. As pH increases above IEP, the polymers in all the micelles are expected to carry increasing net negative charge due to decreasing NR_2_H_2_^+^ content. However, only RCMDX micelles exhibited a continuous transition toward a more negative ζ-potential, while the ζ-potential for the CMDX and MCDX micelles remained largely unchanged, suggesting that enhanced counterion association occurred for the latter two micelles (vide infra). We estimated the polymer-bound charge density in the micellar corona at any given pH using the ellipsoidal micellar structural parameters determined by SAXS analysis (Figure S14). The polymer-bound charge density in the micellar corona was found to increase with increasing change of the solution pH from the IEP. These results indicate that increasing counterion association within the micelles serves to effectively minimize the electrostatic repulsion within the micelles, as pH increases above IEP. In addition, the position of charge groups on the polymer chain modulates the dependencies of the effective surface charges of the micelles on the solution pH.

### Discussion

Three block copolymers used in this study (CMDX, MCDX, and RCMDX) all have a short average chain length (DP_n_ ∼ 25) and an even shorter ionizable C segment (DP_n,C_ < 2). They were obtained by a sequential controlled ring-opening polymerization method (Scheme 1). As a result, all three BCP chains contain the ionizable secondary amine terminus (NR_2_H), whereas a significant fraction of the chains (37 mol %) do not contain the ionizable C monomers due to the statistical nature of the sequential controlled polymerization.^71^ These polypeptoid BCP readily form micelles that are dynamic at elevated temperature (80 °C) and can undergo pH-induced structural reorganization in a broad pH range (2–12) in aqueous solution, owing to the amphoteric nature and low molecular weight of the constituting polypeptoid BCPs.

**Figure 8:**
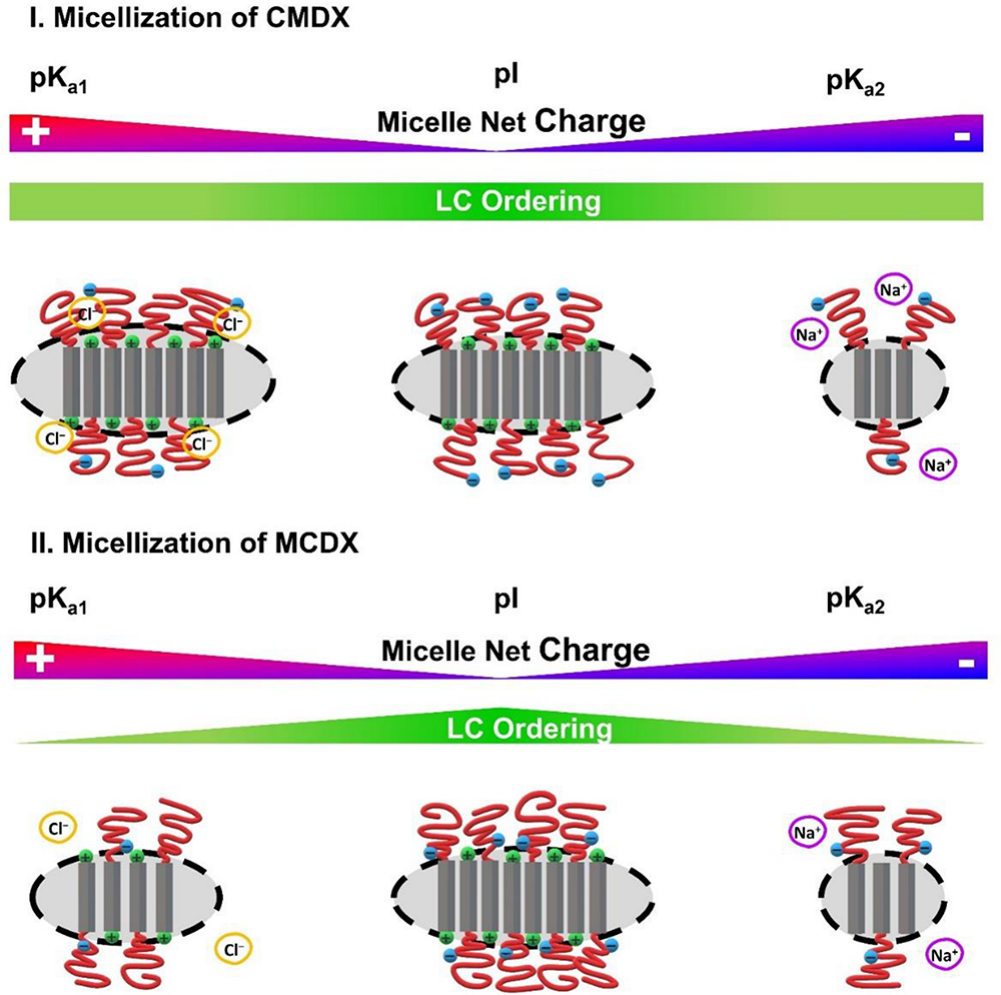
Schematic depiction of the micellar structure of CMDX and MCDX micelles in aqueous solution at different solution pHs. The micellar core (light gray ellipsoids) is comprised of sanidic LC domains formed by the hydrophobic D blocks (dark gray rectangles), and the micellar shell is comprised of the hydrophilic C/M blocks (red lines). The cationic secondary ammonium terminus (NR_2_H_2_^+^) of the hydrophobic blocks and the anionic carboxylate groups (CO_2_^–^) of the C monomer in the hydrophilic blocks are signified by the green and blue spheres, respectively.

Notwithstanding the statistical variation of the composition and chain length inherent to these BCP, changing the anionic C monomer position along the polymer chain has been shown to effectively modulate the micellar size and structure as well as the attendant structural reorganization in response to a change in solution pH. The resulting micellar structures can be understood by considering the interplay of three main interactions: i.e., hydrophobic interaction, liquid crystalline (LC) interaction, and the solvent mediated electrostatic interaction. The hydrophobic interaction provides the driving force for the micellization by minimizing the interfacial tension between the hydrophobic core and solvent. Liquid crystalline interaction between the hydrophobic D segment promotes a compact chain packing into a sanidic LC phase in the micellar core and formation of elongated micelles with a larger aggregation number (Figure 8). The solvation of the charge groups mediates the electrostatic interactions among the polymer-bound charge groups and free counterions and favors loose chain packing in the micelles with smaller aggregation number. While all micellar solutions contain 60 mM NaCl corresponding to a Debye length (κ^–1^) of 12.4 Å (in water, 20 °C), the long-range electrostatic interactions within the micelles are not fully screened at this ionic strength, given that the micellar shell thickness is in the range of 10–20 Å (Figure 5c,d). It is important to note that the LC interaction is coupled to the electrostatic interaction, and the relative contribution of LC and electrostatic interactions to the overall micellar structure can be modulated by the position of the anionic C monomers along the chain (vide infra).

At the IEP, all micelles carry equal amounts of positive (NR_2_H_2_^+^) and negative charges (CO_2_^–^; Figure 8). The intramicellar electrostatic interaction between the polymer bound CO_2_^–^ and NR_2_H_2_^+^ groups render the micelles effectively charge neutral at the IEP, which accounts for the formation of most compact micelles with the highest aggregation number (*n*_A_), radius of gyration (*R*_g_), and molecular ordering (HWHM and *d*-spacing) in the micellar core for all three micelle types in the entire pH range (Figures 4 and 5e,f). As the solution pH departs from the IEP, the net charge in the micelles would increase if the aggregation number were to remain unchanged, resulting in increased electrostatic repulsion in the micelles. To minimize the free energy, the micelles undergo structural reorganization by reducing aggregation number, resulting in increased level of solvation and solvent-mediated counterion association in the micelles as the solution pH is changed from the IEP. This is consistent with the steady decline of *n*_A_ and *R*_g_ and increase of *d*-spacing and HWHM of the liquid crystalline domains as observed for MCDX and RCMDX micelles (Figure 8), supporting the formation of progressively less compact micelles with reduced level of molecular ordering in the micellar core (Figures 4 and 5e,f). For MCDX and RCMDX micelles, it is evident that electrostatic interaction plays a dominant role in modulating their micellar structure in aqueous solution; the liquid crystalline interaction is strongly coupled to the solvent-mediated electrostatic interaction and becomes attenuated with increasing net charge in the MCDX and RCMDX micelles due to the proximity of the anionic C monomer to the micellar core.

Interestingly, for the CMDX micelles, *n*_A_ and *R*_g_ decrease steadily only as the solution pH increases above the IEP and remain comparable without a notable trend of decline for pH values below the IEP (Figures 4 and 8). In addition, CMDX micelles exhibited the most compact and ordered LC domain in the micellar core that is insensitive toward the solution pH change among the three micellar types in the entire ∼2–12 pH range (Figure 5e,f). Moreover, CMDX micelles have the highest aggregation number among the three micellar types for any given solution pH in the entire ∼2–12 pH range. This clearly indicate that the liquid crystalline interaction is stronger and less coupled to the electrostatic interaction in CMDX micelles as compared to the other two micelle types, consistent with the more distant location of the anionic C monomers to the CMDX micellar core. At pH increases above IEP, the electrostatic interaction dominates over LC interaction. CMDX micelles minimize the free energy by reducing the aggregation number and increasing the level of solvation and solvent-mediated counterion association similarly to MCDX and RCMDX micelles. As the pH decreases below IEP, the LC interaction dominates over the electrostatic interaction in their contribution to the free energy of the micelles, resulting in no apparent steady decline of *n*_A_ and *R*_g_.

It is clear that the position of the negative charge (CO_2_^–^) group along the chain can modulate the relative contribution of electrostatic interaction and LC interaction to the micellar structure and the corresponding pH-induced structural reorganization in water. This can be rationalized by considering the difference in the solvation and counterion association of chemically distinct polymer-bound ions at various locations in the micelles. First, the positive charge on the secondary ammonium groups (NR_2_H_2_^+^) is much more delocalized relative to the negative charge on the carboxylate (CO_2_^–^) that is localized on oxygen atoms. The difference in charge density of these organic ions is correlated to much lower hydration energy for the NR_2_H_2_^+^ ions as compared with CO_2_^–^ ions.^72^ As a result, the effect of solvation mediated electrostatic interaction to the micellar structure is expected to be stronger when the overall charge of the micelle is dominated by CO_2_^–^ relative to that of NR_2_H_2_^+^ ions. Second but importantly, the location of the NR_2_H_2_^+^ and CO_2_^–^ groups along the BCP chains also affects their accessibility to solvent.

For CMDX micelles, NR_2_H_2_^+^ groups residing at the more hydrophobic micellar core–shell interface is expected to favor the formation of closely associated ion pairs with the free counterions (Cl^–^) due to restricted access to water.^73^ By contrast, CO_2_^–^ groups residing in a more hydrophilic micellar corona will favor hydration and solvent-mediated association with counterions (Na^+^). The osmotic swelling in the micellar corona leads to effective reduction of the aggregation number of the micelles as the net negative charge increases with increasing pH above IEP. When pH is below IEP, the limited access to water prevents osmotic swelling, and the formation of closely associated ion pairs effectively screens the electrostatic repulsion. The contribution of LC interaction to the overall micellar structure becomes dominant over that of the electrostatic interactions, resulting in no apparent decline of *n*_A_ with decreasing pH below IEP.

For the MCDX micelles, placing the anionic C monomer at the hydrophilic-and-hydrophobic segment junction inhibits the compact liquid crystalline chain packing in the micellar core due to strong hydration of CO_2_^–^ groups.^11^ Consequently, the micellar core surface of MCDX micelles has significantly enhanced access to water as compared to that of CMDX micelles. This allows for solvent-mediated counterion association with NR_2_H_2_^+^ groups residing at the MCDX micellar core surface, resulting in reduced *n*_A_ with decreasing pH below IEP. This behavior is consistent with the finding from a MD simulation where the CO_2_^–^ group of C monomers reoriented themselves to preferentially reside on the micellar surface to be maximally hydrated, resulting in distortion of hydrophilic chain conformation and the micellar shape.^11^

It is interesting to note that a greater difference in *n*_A_ and *R*_g_ between CMDX and MCDX micelles appears at low pHs (2–3) where a less amount of positionally disparate C monomers are ionized, whereas the difference is much smaller toward the high pH range (10–12) where the C monomers are fully ionized. At high pH range (above IEP), the increased solvent mediated counterion association causes osmotic swelling of the micelles. As the counterions (Na^+^) are loosely associated with the CO_2_^–^ groups on C monomers, osmotic swelling of the micellar corona is not restricted to the immediate surroundings of the C monomers. As a result, the effect of osmotic swelling on the micellar structure is less sensitive to the location of the C monomers in the micellar corona, which accounts for the diminished difference in *n*_A_ and *R*_g_ between CMDX and MCDX micelles as the pH increases toward higher ends (10–12). At the low pH end (2–3) where there are fewer ionized C monomers, the position of anionic C monomers modulates the solvent accessibility to the micellar core–coronal interface where NR_2_H_2_^+^ resides, thus influencing the relative strength and contribution of LC interaction and electrostatic interaction to the overall micellar structure. For CMDX micelles, where the C monomers are located far from the micellar core, the LC interaction contributes more to the micellar structure than the electrostatic interaction, thereby resulting in larger *n*_A_ and *R*_g_. By contrast, for MCDX micelles where the C monomers are proximate to the micellar core, the LC interaction is weakened, and the electrostatic interaction dominates, leading to smaller *n*_A_ and *R*_g_. One would reason at very low pHs (≪2–3), i.e., several pH units below p*K*_a,1_ where no C monomer is ionized and the electrostatic interaction is absent, CMDX and MCDX micelles should have the same equilibrium micellar structure with identical *n*_A_ and *R*_g_, resulted from balancing the LC interaction with the excluded volume interaction among neutral chains. This aspect will be investigated in future.

Considering the above contributing interactions, one would expect the chain packing of the RCMDX micelles to be between that in the CMDX and MCDX micelles, given that the negative charges are randomly distributed along the hydrophilic segment of RCMDX micelles. But unexpectedly, RCMDX micelles exhibited the lowest *n*_A_ among all three micellar types in the entire pH range. Several factors may have contributed to this unexpected trend. First, RCMDX polymers have a slightly lower hydrophobic D monomer and higher ionizable C monomer content relative to CMDX and MCDX micelles (Table S1). The enhanced electrostatic interaction and reduced hydrophobic and LC interactions can account for the smaller *n*_A_ for the RCMDX micelles. Second, the ionizable C monomer distribution along the RCMDX chains is different from that of the other two polymers. A significant fraction of RCMDX polymers can have nonsequential distribution of multiple C monomers, i.e., multiple isolated ionization sites along the hydrophilic block, in contrast to CMDX and MCDX polymers where multiple C monomers must present at sequential locations along the chains. Nonsequential distribution of multiple ionization sites along the chain tend to promote greater extent of ionization and less extent of counterion association relative to the sequential distribution of multiple ionization sites, noting that Bjerrum length (=7.1 Å in water, at 20 °C) is more than doubling the end-to-end distance of a repeating unit (ca. 3 Å) of the polypeptoid BCPs in an extended conformation.^55^ As a result, the enhanced electrostatic interaction may also contribute to a reduced *n*_A_ for RCMDX micelles relative to the other two micelle types.

## Conclusions

We have synthesized amphoteric polypeptoid block copolymers where one ionizable monomer is positionally fixed along the polymer chain while the position of the other ionizable monomer along the chains is varied by a sequential ring-opening polymerization method. The presence of ionizable monomers with opposite charges on the amphoteric BCP chains has resulted in micellar assemblies that can undergo structural reorganization over a wide pH range (2–12) in an aqueous solution. The position of the ionizable monomer along the hydrophilic segment has been shown to influence their pH-dependent micellar structures in a manner that is notably different from when the ionizable monomers are randomly distributed along the segment, highlighting the role of the charge pattern in modulating the solution assemblies of polymers. The study demonstrated that encoding electrostatic interactions in the block sequence of multiblock copolymers can effectively modulate their supramolecular assemblies in solution and the corresponding structural reorganization in response to solution pH change, notwithstanding the statistical variation of composition and chain length inherent to the polymers. Charge pattern represents a potentially useful design parameter toward tailored molecular assemblies for different targeted applications.

## Supporting Information Available

Polymer characterization data (^1^H NMR spectra, FTIR spectra, SEC-MALS-dRI chromatograms), CMC determination, p*K*_a_ titration, and ζ-potential measurements of the micellar solutions, DSC thermograms, SANS, SAXS, and DLS analyses for the CMDX, MCDX, and RCMDX, function form of the core–shell ellipsoidal model, estimation of polymer bound charge density in the micellar corona (PDF)
