# Supplementary material for: pH-Dependent Solution Micellar Structure of Amphoteric Polypeptoid Block Copolymers with Positionally Controlled Ionizable Sites
Source: Biomacromolecules. 2023 Jul 21;24(8):3700–15. doi: 10.1021/acs.biomac.3c00407 (PMC10428163; doi:10.1021/acs.biomac.3c00407)
Supplement: Supplementary file 1 — bm3c00407_si_001.pdf [file bm3c00407_si_001.pdf]

Supplementary Information

pH-Dependent Solution Micellar Structure of Amphoteric Polypeptoid Block Copolymers with Positionally Controlled Ionizable Sites

Meng Zhang,[a] Yun Liu,[b] Xiaobing Zuo,[c] Shuo Qian,[d] Sai Venkatesh Pingali,[e] Richard E. Gillilan,[f] Qingqiu Huang,[f] and Donghui Zhang[a,]*

[a.] *Department of Chemistry and Macromolecular Studies Group, Louisiana State University, Baton Rouge, LA 70803, United States*

[b.] *Center for Neutron Research, National Institute of Standards and Technology, Gaithersburg, MD 20899, United States*

[c.] *X-ray Science Division, Argonne National Laboratory, Lemont, IL 60439, United States*

[d.] *Neutron Scattering Division and Second Target Station, Oak Ridge National Laboratory, Oak Ridge, TN 37831, United States*

[e.] *Neutron Scattering Division, Oak Ridge National Laboratory, Oak Ridge, TN 37831, United States*

[f.] *MacCHESS (Macromolecular Diffraction Facility at CHESS), Cornell University, Ithaca, NY 14850, United States*

Corresponds to: dhzhang@lsu.edu

Guinier Analysis:[S1]

$\ln(I(Q)) = \ln\left(\phi(\rho_{solvent} - \rho_{polymer})^2 V_m\right) - \frac{R_g{}^2 Q^2}{3}$ (Eq. S1)

$\ln(I(Q)) = \ln\left(\frac{c n_A (\Delta\rho)^2 M_w}{N_A \rho^2}\right) - \frac{R_g{}^2 Q^2}{3}$ (Eq. S2)

ϕ: volume fraction

V_m: volume of polymer

n_A: aggregation number

$\rho_{solvent}$: solvent scattering length density

$\rho_{polymer}$: polymer scattering length density

R_g: radius of gyration

N_A: Avogadro constant

ρ: polymer mass density

M_w: polymer molecular weight

c: mass concentration

$\Delta\rho$: scattering length density difference ($\rho_{polymer} - \rho_{solvent}$)

(Note that the neutron scattering length densities (SLDs) of hydrophobic block and hydrophilic block of CMDX, MCDX and RCMDX micelles were determined from the corresponding polymer mass densities).[S2-S4]

Core-Shell Ellipsoidal Model: [S5-S6]

$I(q, \alpha) = \frac{scale}{V} F^2(q) + background$ (Eq. S3)

$F(q, \alpha) = f(q, R_e, R_e \cdot x_{core}, \alpha) + f(q, R_e + t_{shell}, R_e \cdot x_{core} + t_{shell} \cdot x_{polarshell}, \alpha)$ (Eq. S4)

$f(q, R_e, R_p, \alpha) = \frac{3\Delta\rho V(\sin[qr(R_p, R_e, \alpha)] - \cos[qr(R_p, R_e, \alpha)])}{[qr(R_p, R_e, \alpha)]^3}$ (Eq. S5)

$r(R_p, R_e, \alpha) = [R_e^2 sin^2\alpha + R_p^2 cos^2\alpha]^{1/2}$ (Eq. S6)

α: angle between the axis of the ellipsoid and $\vec{q}$

V: volume of ellipsoid: $V = \frac{4}{3}\pi R_p R_e^2$

R_p: polar radius along the rotational axis of the ellipsoid

R_e: equatorial radius perpendicular to the rotational axis of the ellipsoid

t_{shell}: thickness of the shell at the equator

Δρ: scattering length density difference, either $(\rho_{core} - \rho_{shell})$ or $(\rho_{shell} - \rho_{solvent})$

(Note that the X-ray scattering length densities (SLDs) of hydrophobic block and hydrophilic block of CMDX, MCDX and RCMDX micelles can be determined from corresponding polymer mass densities).[S2-S4]

Estimation of Polymer Bound Charge Density in the Micellar Corona

The polymer bound charge densities in the micelles can be estimated from the ionization states of the carboxyl (COOH) and amine groups (NR_2H) and the micellar corona volume at a given solution pH as shown below (Eq. S7-S12). The micellar corona volume (V_{corona}) was calculated using the micellar dimensions determined by SAXS analyses using the core-shell ellipsoidal model. The aggregation number (n_A) of the micelles was obtained from SANS analyses. pK_a values of carboxyl and ammonium groups in the micelles were determined by titration.

$pH = pK_a + log\frac{\alpha}{1-\alpha}$ (Eq. S7)

$\alpha_1 = \frac{1}{10^{pK_{a1}-pH}+1}$ (Eq. S8)

$\alpha_2 = \frac{1}{10^{pK_{a2}-pH}+1}$ (Eq. S9)

$polymer\ bound\ charge\ density\ in\ the\ micelles = \frac{[1\times\alpha_1 - 1\times(1-\alpha_2)]\cdot n_A}{V_{corona}}$ (Eq. S10)

α_1: percentage of ionization of the carboxyl group (COOH) in a polymer chain

α_2: percentage of ionization of the amine group (NR_2H) in a polymer chain

$pK_{a,1}$: pK_a value of carboxyl groups (COOH) in the micelle

$pK_{a,2}$: pK_a value of ammonium groups ($NR_2H_2^+$) in the micelle

n_A: aggregation number

V_{corona}: volume of ellipsoid-shaped micellar corona = $\frac{4\pi[(R_2+T_2)^2\cdot(R_1+T_1)-(R_2^2\cdot R_1)]}{3}$ (Eq. S11)

R_1 and R_2: core radius of the long and short axis of the ellipsoidal micelles, respectively

T_1 and T_2: shell thickness of the long and short axis of the ellipsoidal micelles

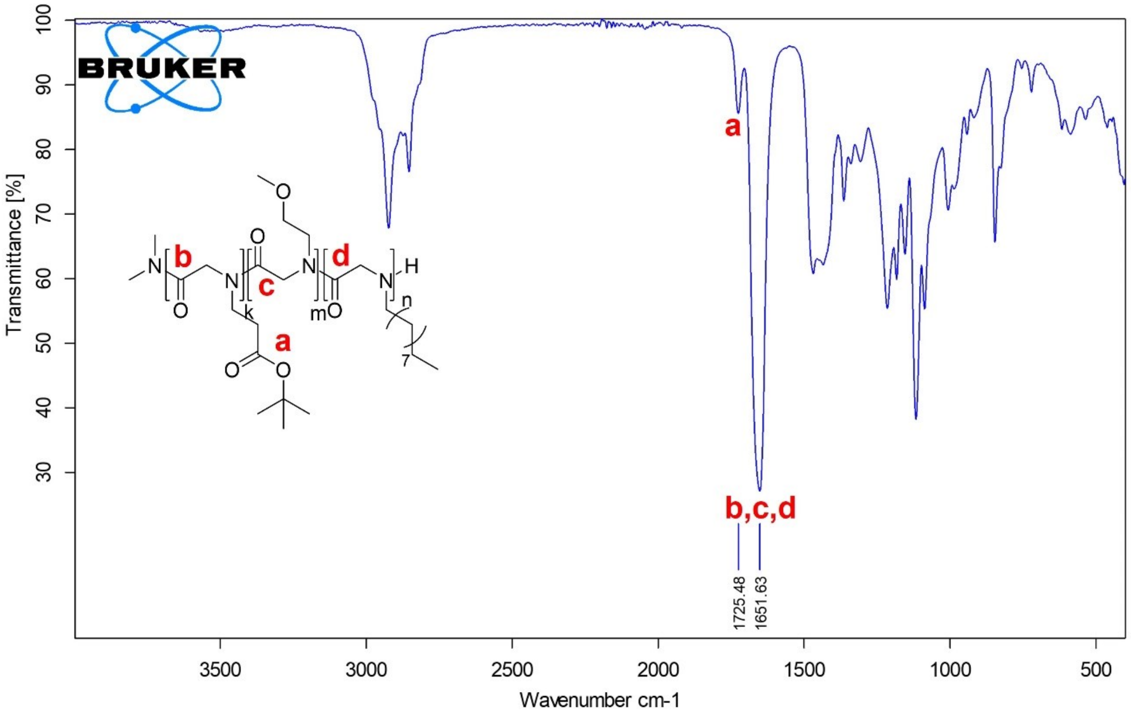

Figure S1. Representative FTIR spectrum of poly(*N*-(3-*tert*-butoxy-3-oxopropyl) glycine)-*b*-poly(*N*-2-methoxyethyl glycine)-*b*-poly(*N*-decyl glycine) block copolymer (CMDX precursor).

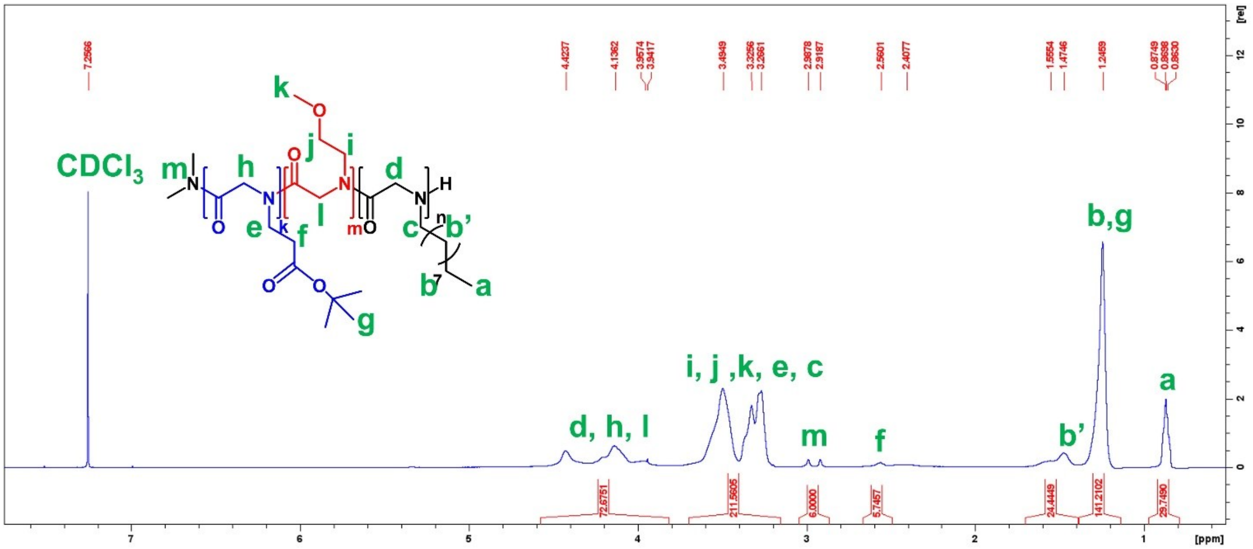

Figure S2. ^{1}H NMR spectrum of poly(*N*-(3-*tert*-butoxy-3-oxopropyl) glycine)-*b*-poly(*N*-2-methoxyethyl glycine)-*b*-poly(*N*-decyl glycine) block copolymer (CMDX precursor).

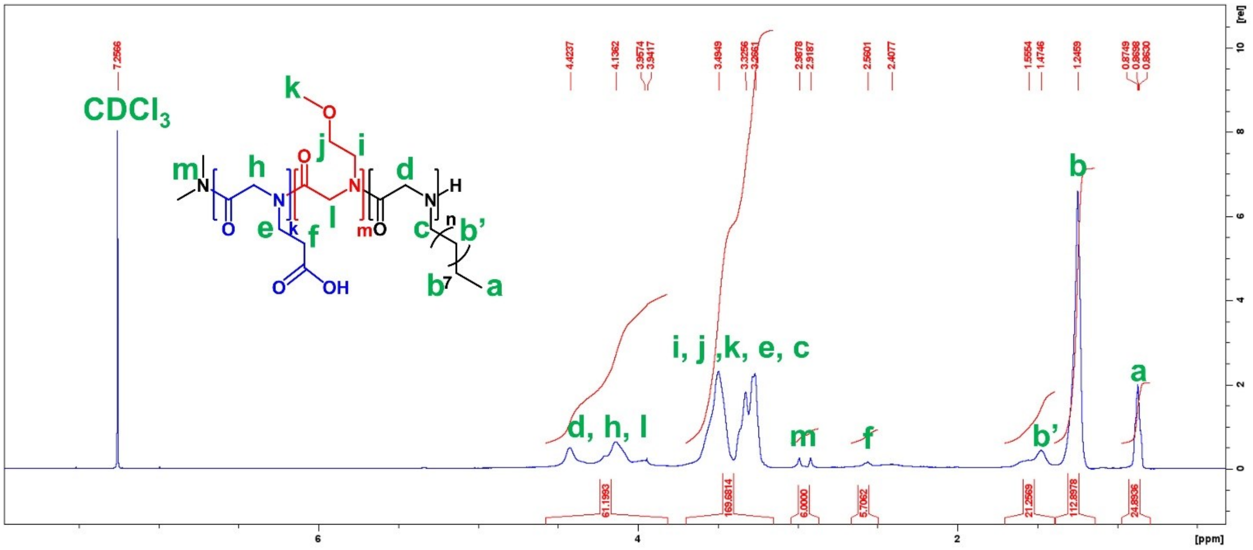

Figure S3. ^{1}H NMR spectrum of poly(*N*-2-carboxyethyl glycine)-*b*-poly(*N*-2-methoxyethyl glycine)-*b*-poly(*N*-decyl glycine) block copolymer (CMDX).

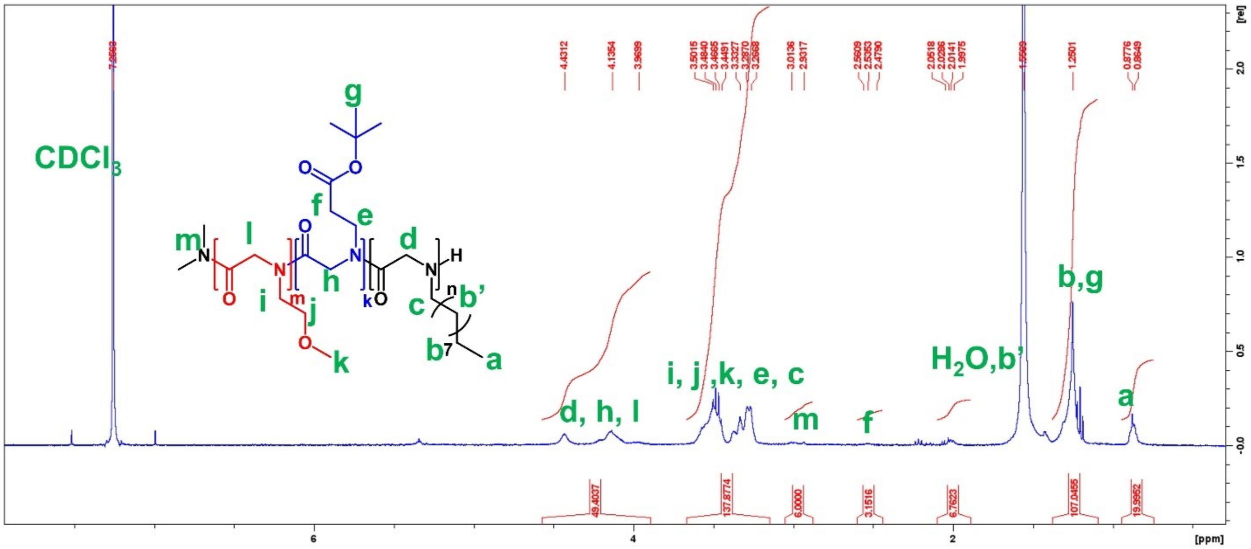

Figure S4. ^{1}H NMR spectrum of poly(*N*-2-methoxyethyl glycine)-*b*-poly(*N*-(3-*tert*-butoxy-3-oxopropyl) glycine)-*b*-poly(*N*-decyl glycine) block copolymer (MCDX precursor).

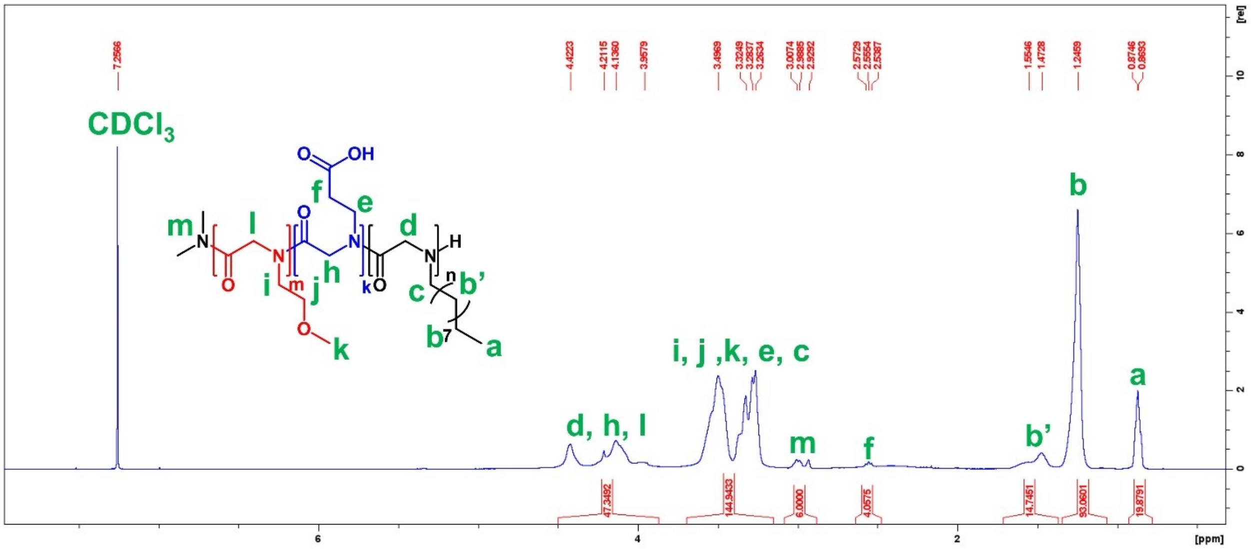

Figure S5. ^{1}H NMR spectrum of poly(*N*-2-methoxyethyl glycine)-*b*-poly(*N*-2-carboxyethyl glycine)-*b*-poly(*N*-decyl glycine) block copolymer (MCDX).

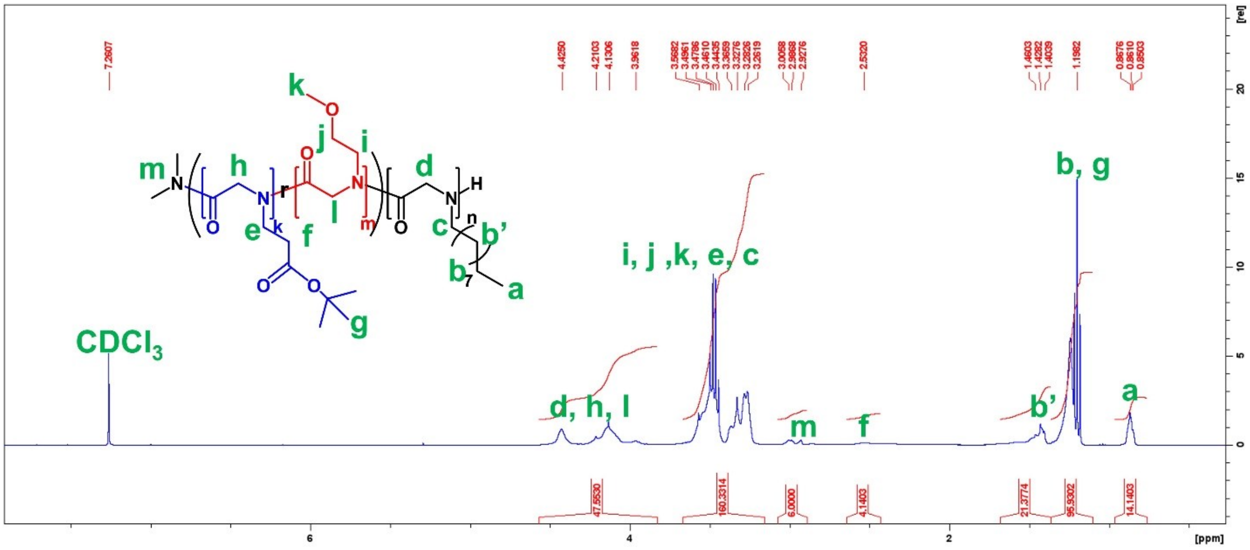

Figure S6. ^{1}H NMR spectrum of (poly(*N*-(3-*tert*-butoxy-3-oxopropyl) glycine)-*r*-poly(*N*-2-methoxyethyl glycine))-*b*-poly(*N*-decyl glycine) block copolymer (RCMDX precursor).

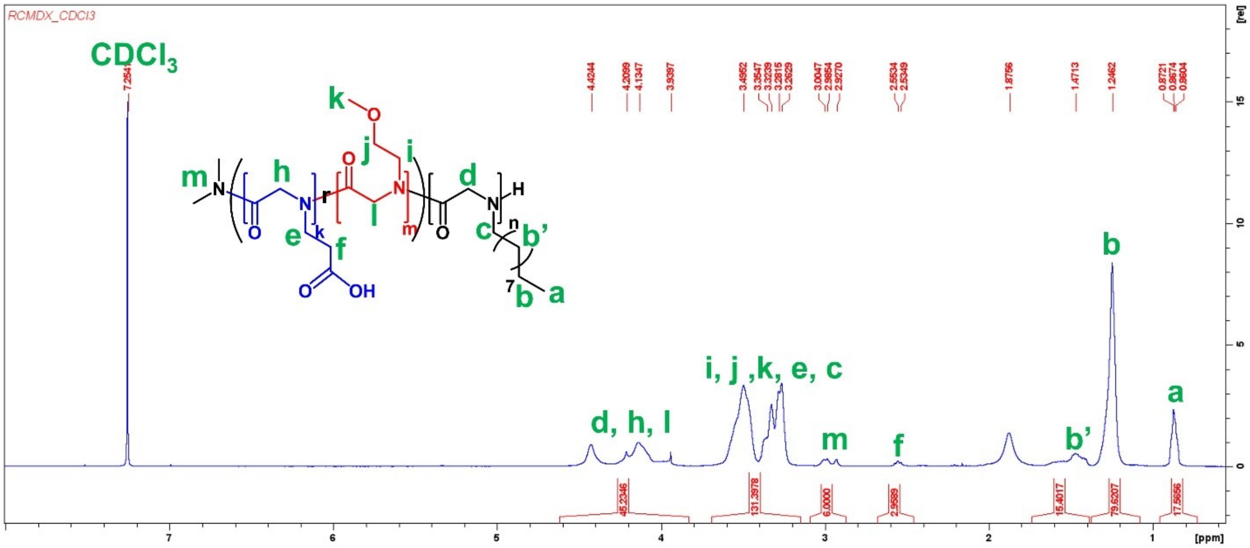

Figure S7. ^{1}H NMR spectrum of poly(*N*-2-carboxyethyl glycine)-*r*-poly(*N*-2-methoxyethyl glycine))-*b*-poly(*N*-decyl glycine) block copolymer (RCMDX).

Table S1. Respective volume fraction of the ionizable (C), non-ionic hydrophilic (M) and hydrophobic segment (D+X) in CMDX, MCDX and RCMDX block copolymers

Polymer composition	Volume Fraction [a]		
	C monomer	M monomer	D monomer
$C_{1.2}M_{17}D_{4.7}X$	5.2%	58%	37%
$M_{18}C_{1.1}D_{4.7}X$	4.6%	60%	36%
$RC_{1.9}M_{18}D_{3.9}X$	8.2%	61%	32%

[a] Volume fraction of C, M, D monomer (or repeating unit) in the respective BCP was calculated based on the polymer composition and densities of C, M and D monomer (or repeating unit) that were 0.996, 1.18 and 0.95 g/cm^3, respectively. [S2-S4]

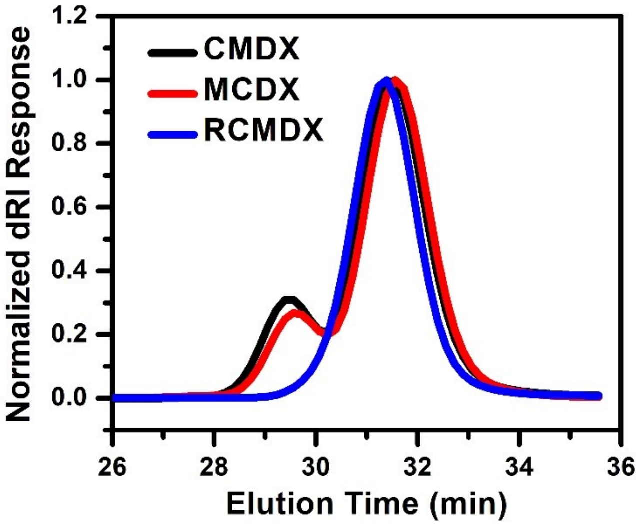

Figure S8. SEC-dRI chromatograms of CMDX (black line), MCDX (red line) and RCMDX (blue line) block copolymers. The small shoulders observed at short elution time (28-30 min) for CMDX and MCDX samples are due to polymer aggregation in the HFIP/CF_3CO_2K (3 mg/mL) solvent used in the SEC analyses.

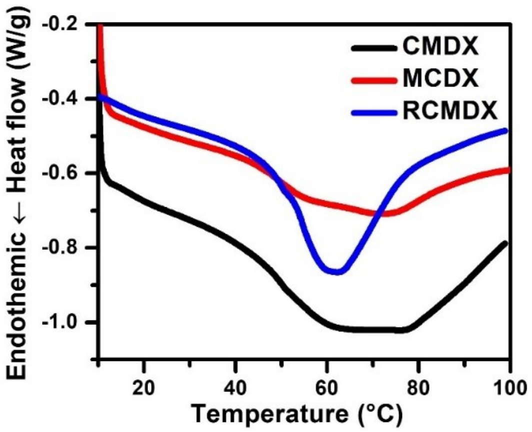

Figure S9. DSC thermograms of the CMDX (black line), MCDX (red line) and RCMX (blue line) block copolymers in the first heating cycle. The polymers were obtained by lyophilization of the corresponding micellar solutions.

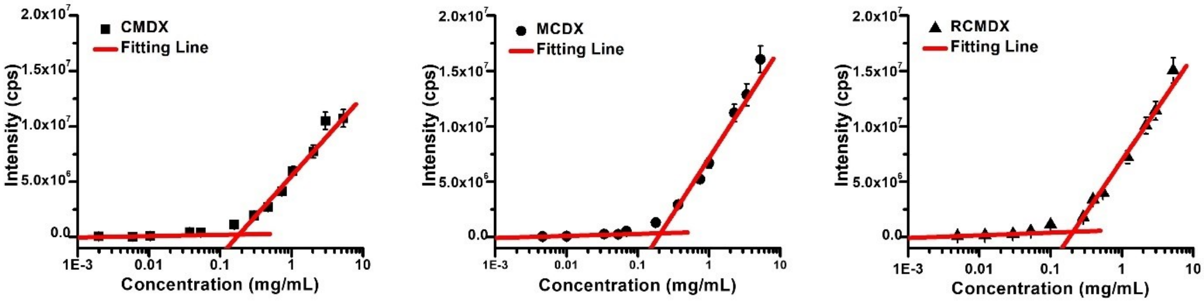

Figure S10. CMC measurements of CMDX, MCDX and RCMDX block copolymer solutions with a constant 60 mM NaCl concentration at 25 °C.

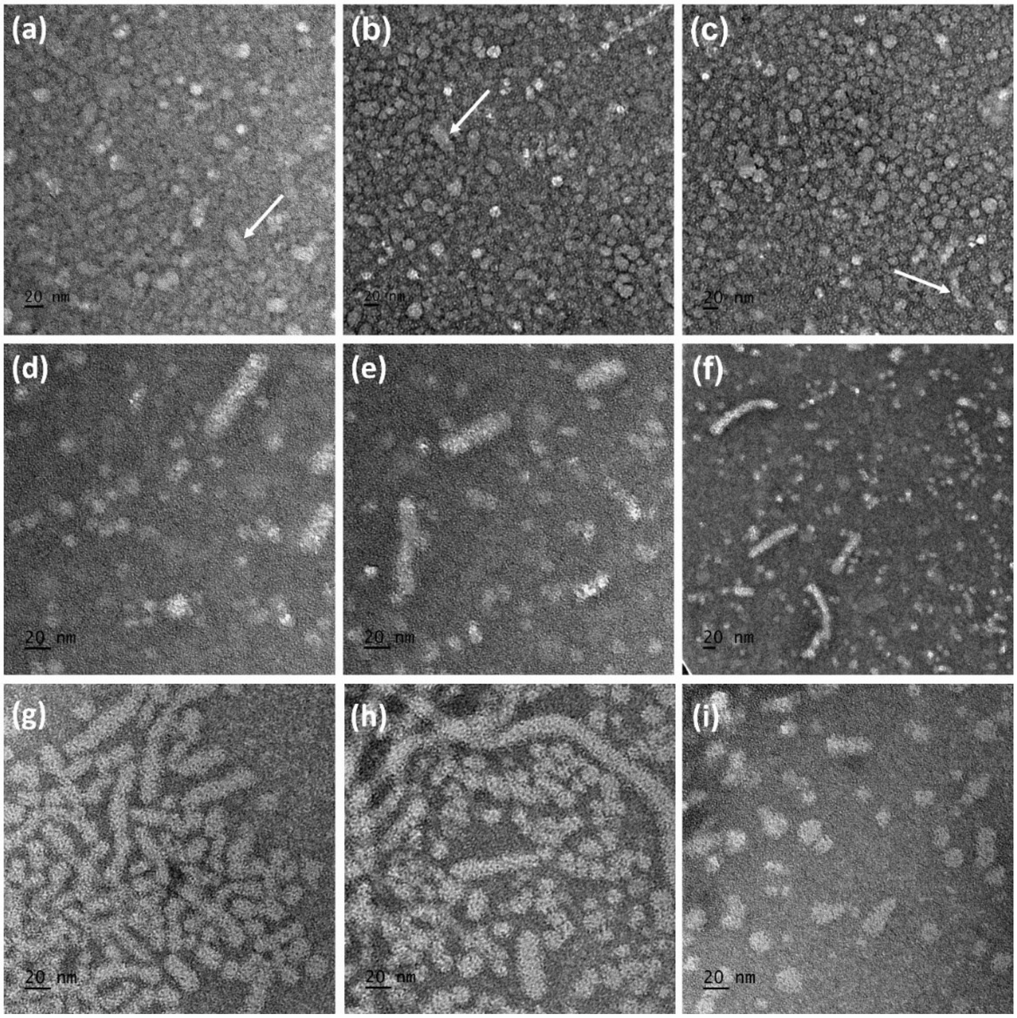

Figure S11. TEM images of the CMDX (a-c), MCDX (d-f) and RCMDX micelles (g-i) where occasional ellipsoidal micelles (marked with arrow) or notably elongated micelles were observed. The scale bar is 20 nm.

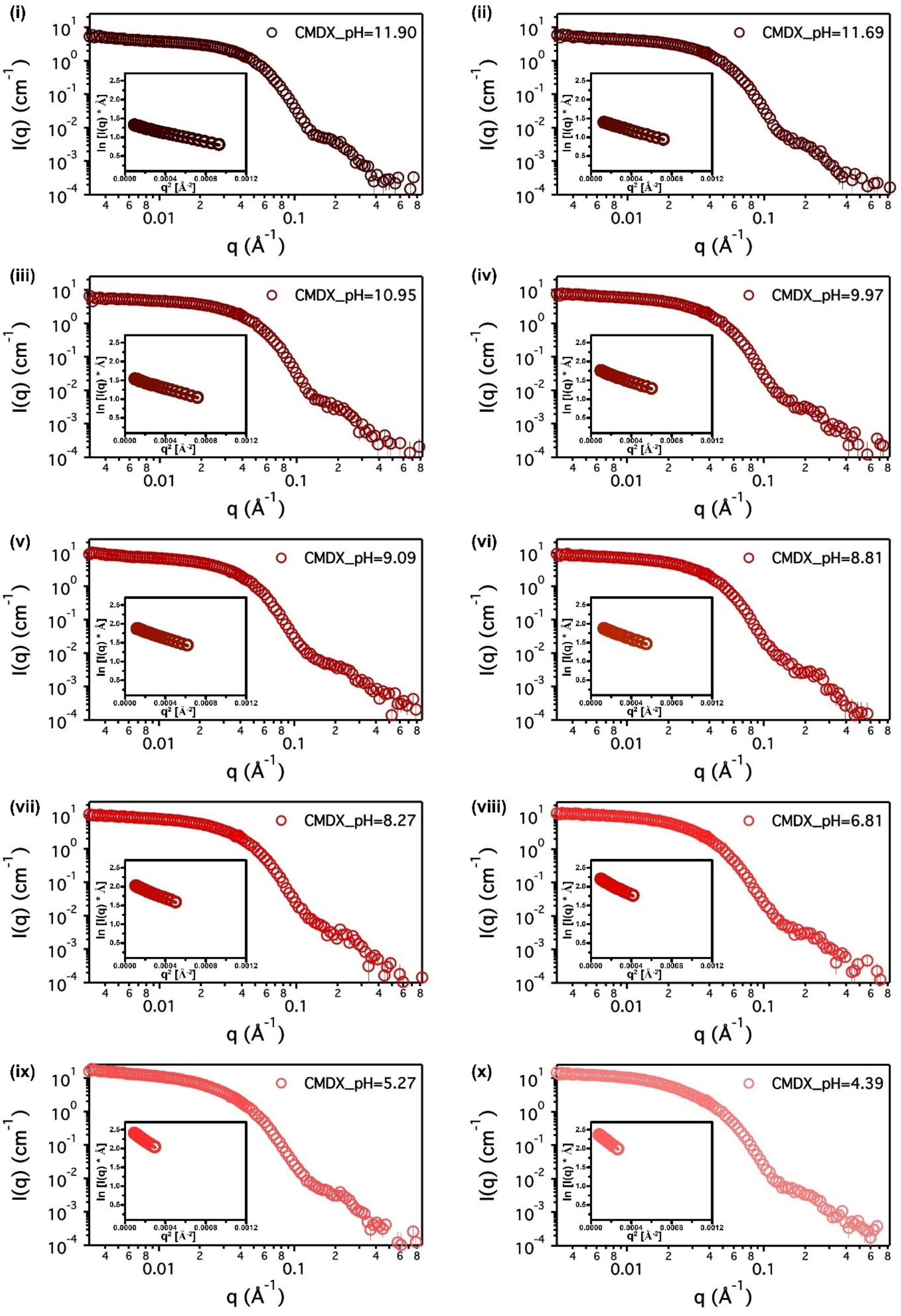
(i)
CMDX_pH=11.90
(ii)
CMDX_pH=11.69
(iii)
CMDX_pH=10.95
(iv)
CMDX_pH=9.97
(v)
CMDX_pH=9.09
(vi)
CMDX_pH=8.81
(vii)
CMDX_pH=8.27
(viii)
CMDX_pH=6.81
(ix)
CMDX_pH=5.27
(x)
CMDX_pH=4.39
I(q) (cm-1)
q (Å-1)
ln [I(q) * Å]
q2 [Å-2]

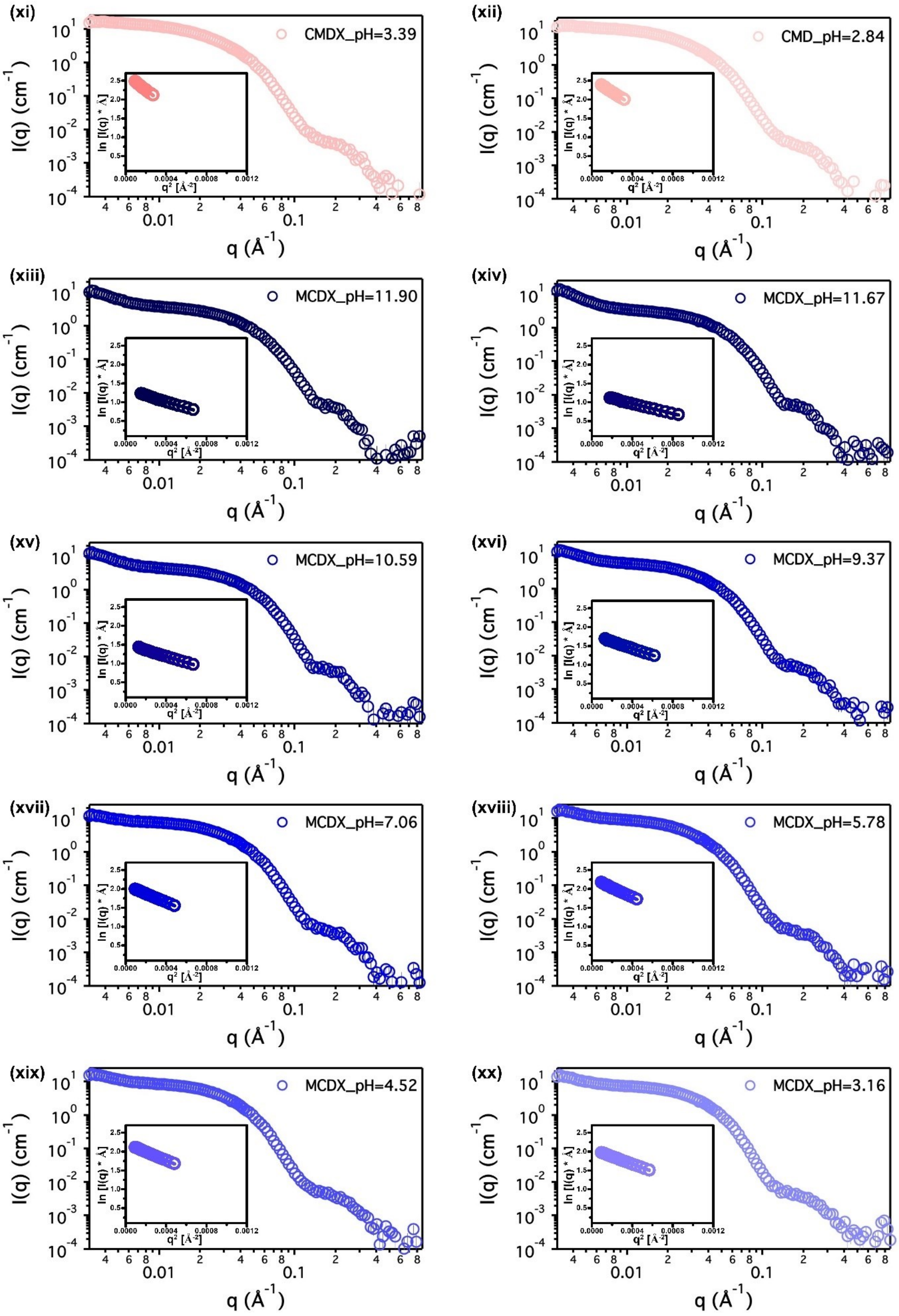
(xi)
CMDX_pH=3.39
(xii)
CMD_pH=2.84
(xiii)
MCDX_pH=11.90
(xiv)
MCDX_pH=11.67
(xv)
MCDX_pH=10.59
(xvi)
MCDX_pH=9.37
(xvii)
MCDX_pH=7.06
(xviii)
MCDX_pH=5.78
(xix)
MCDX_pH=4.52
(xx)
MCDX_pH=3.16
I(q) (cm^{-1})
q (Å^{-1})
ln [I(q) * Å]
q^2 [Å^{-2}]

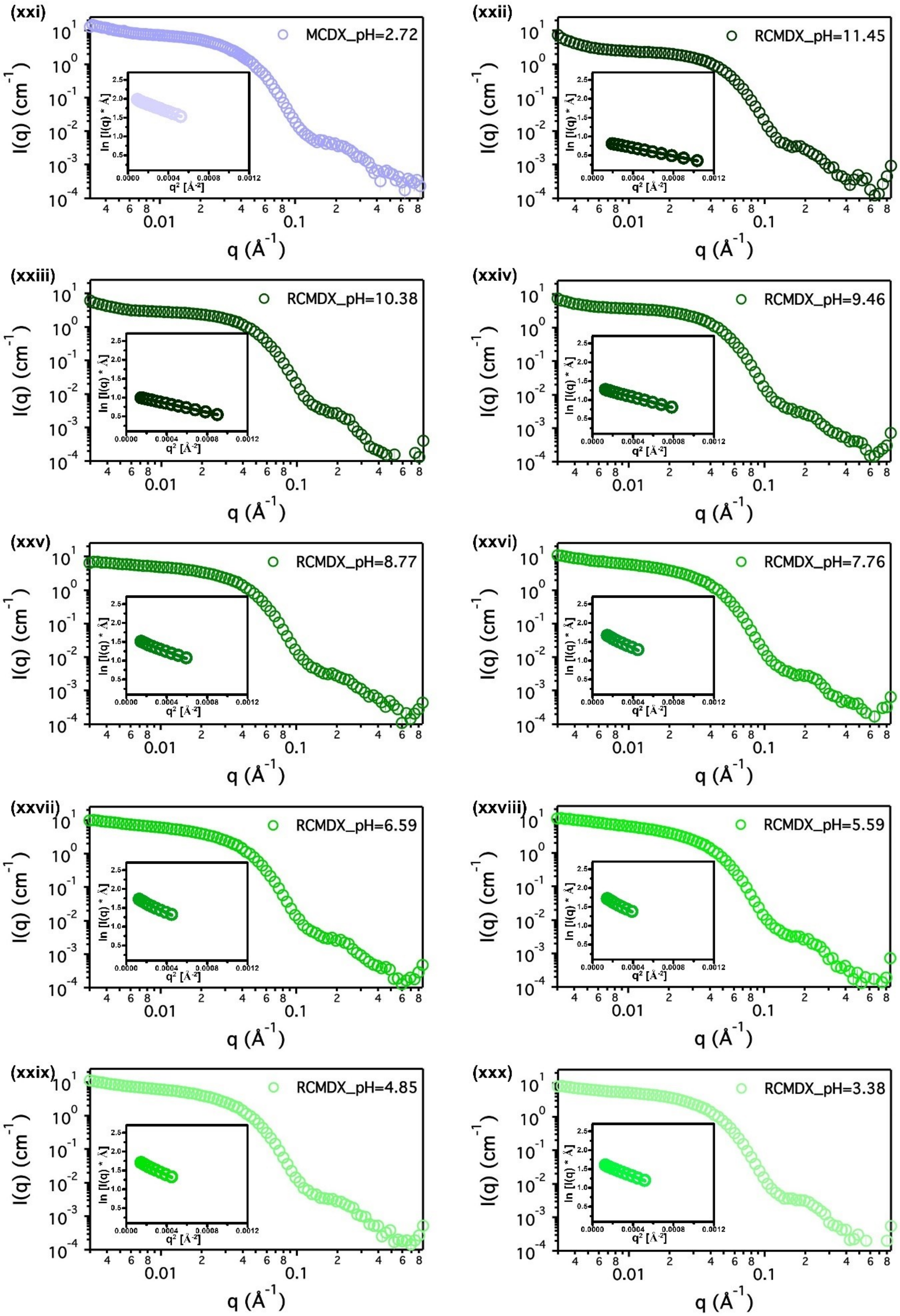
(xxi)
MCDX_pH=2.72
(xxii)
RCMDX_pH=11.45
(xxiii)
RCMDX_pH=10.38
(xxiv)
RCMDX_pH=9.46
(xxv)
RCMDX_pH=8.77
(xxvi)
RCMDX_pH=7.76
(xxvii)
RCMDX_pH=6.59
(xxviii)
RCMDX_pH=5.59
(xxix)
RCMDX_pH=4.85
(xxx)
RCMDX_pH=3.38
I(q) (cm^{-1})
q (Å^{-1})
ln [I(q) * Å]
q^2 [Å^{-2}]

(xxxi)

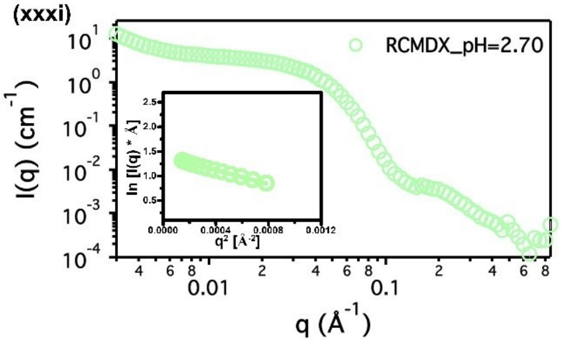

Figure S12. SANS intensity profiles and Guinier plots (insets) for CMDX (red open circles and lines), MCDX (blue open circles and lines), and RCMDX (green open circles and lines) at pH ~2-12 range (measurement condition: [polymer] = 5.0 mg/mL, [NaCl] = 60 mM, in D_2O, at 20 °C).

Table S2. Aggregation number (n_A) and radius of gyration (R_g) of CMDX, MCDX and RCMDX micelles at different solution pHs determined by Guinier analysis of SANS profiles.

Sample Name	pH [a]	n_A	R_g (Å)
**CMDX-1 (SANS)**	2.84	189.1±0.7	71.9±0.4
**CMDX-2 (SANS)**	3.39	214.9±1	79.1±0.6
**CMDX-3 (SANS)**	4.39	186.2±0.8	78.2±0.4
**CMDX-4 (SANS)**	5.27	195.2±1	74.5±0.6
**CMDX-5 (SANS)**	6.81	151.2±1	64.0±0.4
**CMDX-6 (SANS)**	8.27	124.5±0.6	57.2±0.4
**CMDX-7 (SANS)**	8.81	108.3±0.5	53.8±0.3
**CMDX-8 (SANS)**	9.09	107.3±0.4	51.9±0.3
**CMDX-9 (SANS)**	9.97	93.2±0.4	53.1±0.4
**CMDX-10 (SANS)**	10.95	73.8±0.3	48.6±0.3
**CMDX-11 (SANS)**	11.69	66.1±0.2	48.3±0.2
**CMDX-12 (SANS)**	11.90	58.3±0.2	43.0±0.3
**MCDX-1 (SANS)**	2.72	113.8±0.3	55.2±0.3
**MCDX-2 (SANS)**	3.16	114.5±0.2	54.6±0.2
**MCDX-3 (SANS)**	4.52	133.5±0.3	58.3±0.2
**MCDX-4 (SANS)**	5.78	142.8±0.5	61.8±0.3

**MCDX-5 (SANS)**	7.06	118.4±0.3	58.2±0.2
**MCDX-6 (SANS)**	9.37	87.4±0.3	52.2±0.3
**MCDX-7 (SANS)**	10.59	66.6±0.2	50.0±0.3
**MCDX-8 (SANS)**	11.67	49.8±0.2	44.9±0.3
**MCDX-9 (SANS)**	11.90	55.9±0.3	49.9±0.4
**RCMDX-1 (SANS)**	2.70	61.9±0.2	46.2±0.3
**RCMDX-2 (SANS)**	3.38	85.2±0.4	55.9±0.4
**RCMDX-3 (SANS)**	4.85	99.6±0.8	61.4±0.7
**RCMDX-4 (SANS)**	5.59	102.5±1	64.6±1
**RCMDX-5 (SANS)**	6.59	98.8±1	61.7±0.9
**RCMDX-6 (SANS)**	7.76	95.2±1	61.2±0.9
**RCMDX-7 (SANS)**	8.77	78.1±0.6	54.1±0.6
**RCMDX-8 (SANS)**	9.46	59.1±0.1	46.2±0.1
**RCMDX-9 (SANS)**	10.38	44.6±0.05	42.1±0.1
**RCMDX-10 (SANS)**	11.45	37.9±0.04	40.3±0.1

[a.] pH values of all micellar solutions in D_2O was obtained by using the following relation: pH = pH* ×0.929 + 0.42 where pH* is the direct reading of a H_2O-calibrated pH meter in a D_2O solution.[S7]

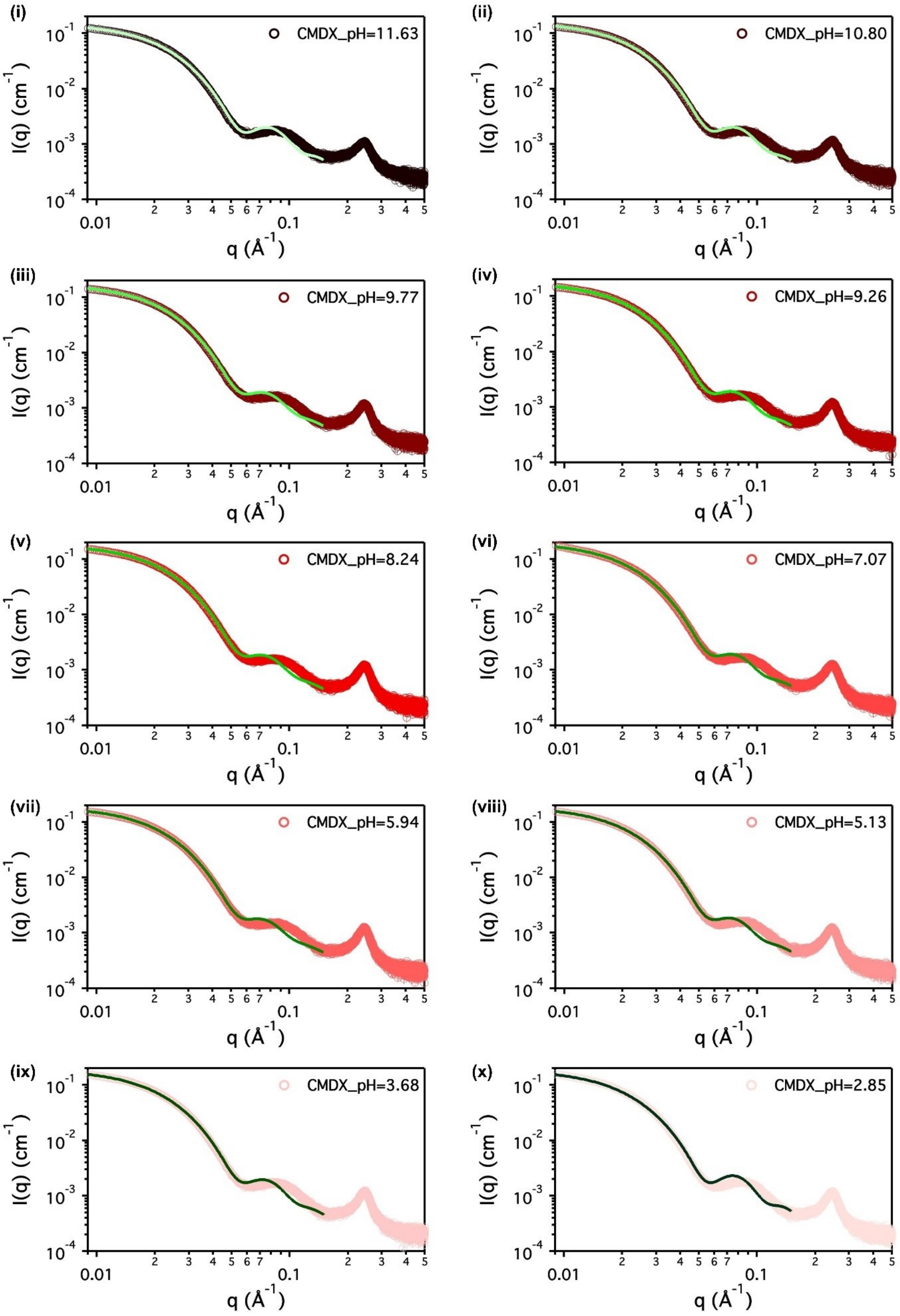
(i)
CMDX_pH=11.63
(ii)
CMDX_pH=10.80
(iii)
CMDX_pH=9.77
(iv)
CMDX_pH=9.26
(v)
CMDX_pH=8.24
(vi)
CMDX_pH=7.07
(vii)
CMDX_pH=5.94
(viii)
CMDX_pH=5.13
(ix)
CMDX_pH=3.68
(x)
CMDX_pH=2.85
I(q) (cm^{-1})
q (Å^{-1})
0.01
0.1
10^{-1}
10^{-2}
10^{-3}
10^{-4}

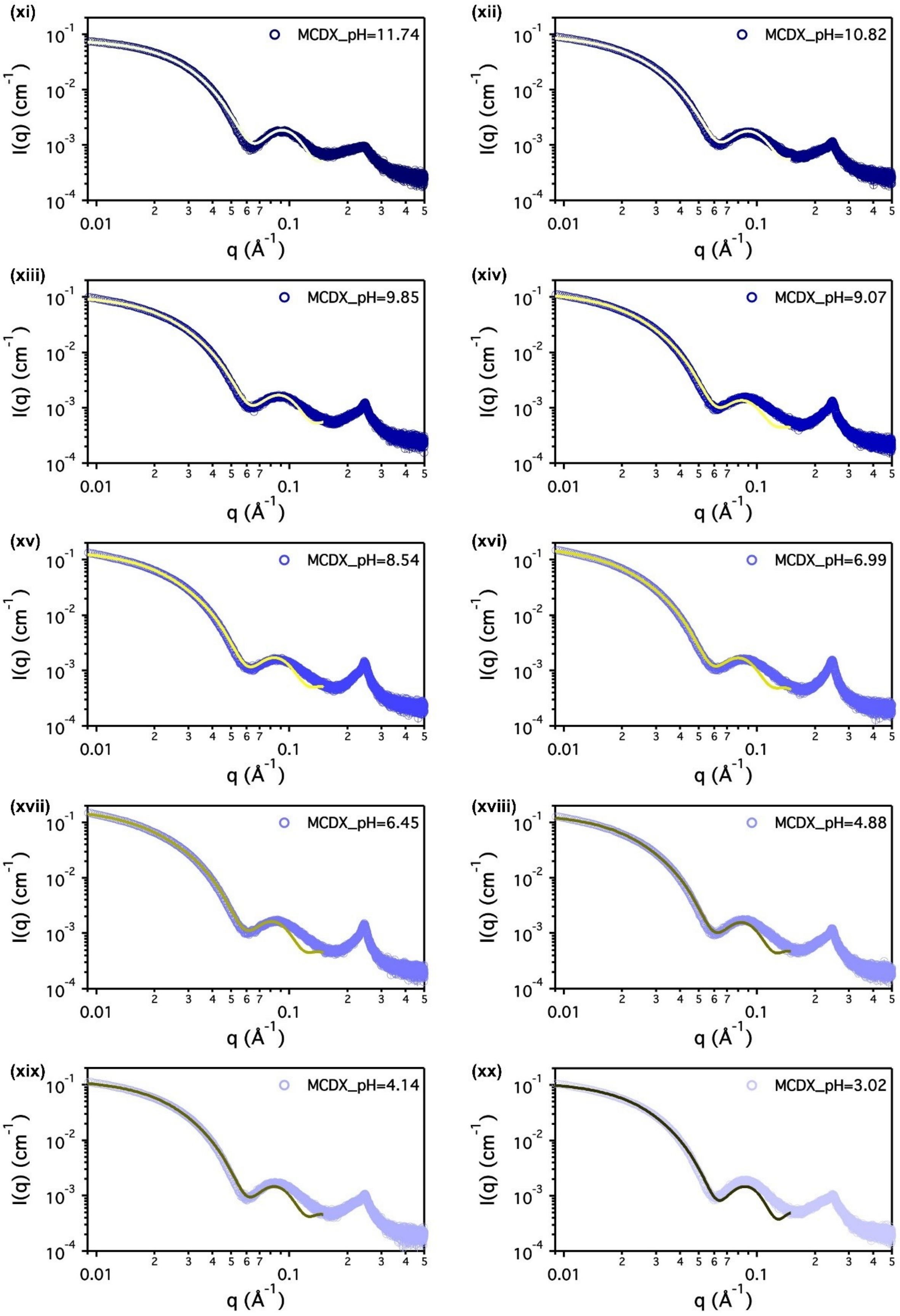
(xi)
MCDX_pH=11.74
(xii)
MCDX_pH=10.82
(xiii)
MCDX_pH=9.85
(xiv)
MCDX_pH=9.07
(xv)
MCDX_pH=8.54
(xvi)
MCDX_pH=6.99
(xvii)
MCDX_pH=6.45
(xviii)
MCDX_pH=4.88
(xix)
MCDX_pH=4.14
(xx)
MCDX_pH=3.02
I(q) (cm^{-1})
q (Å^{-1})
10^{-1}
10^{-2}
10^{-3}
10^{-4}
0.01
0.1

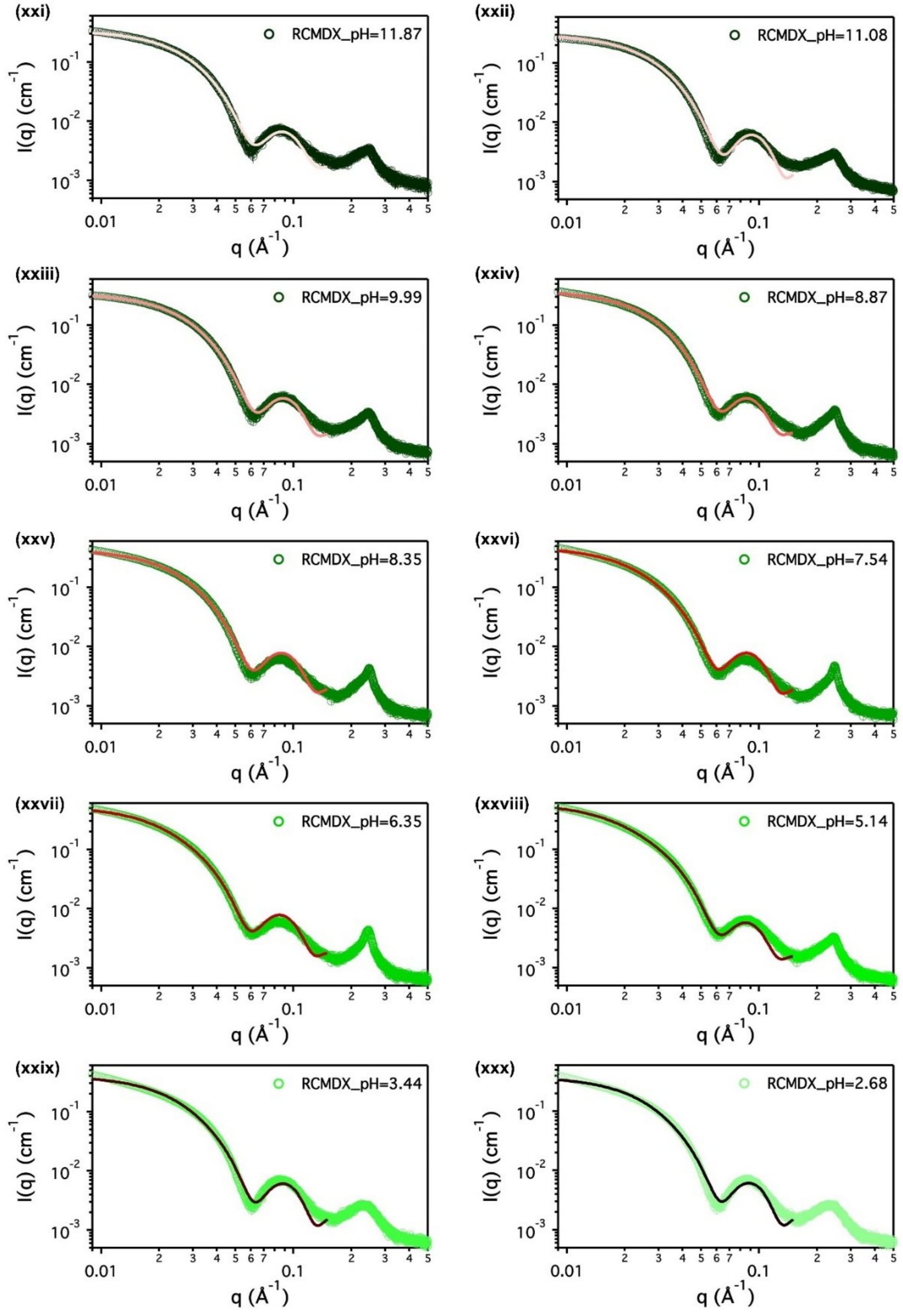

Figure S13. SAXS intensity profiles and fitting curves (core-shell ellipsoidal model) for CMDX (red open circles and lines), MCDX (blue open circles and lines), and RCMDX (green open circles and lines) at pH ~2-12 range (measurement condition: [polymer] = 5.0 mg/mL, [NaCl] = 60 mM, in H_2O, at 20 °C).

Table S3. Structural parameters of CMDX, MCDX and RCMDX micelles at different solution pHs obtained by SAXS analyses using core-shell ellipsoidal model.

Sample Name	pH	R_1 (Å)	R_2 (Å)	T_1 (Å)	T_2 (Å)	SLD_{core}[a] (10^{-6}/Å^2)	SLD_{corona}[a] (10^{-6}/Å^2)
**CMDX-1 (SAXS)**	2.85	42.33 ±0.02	127.53 ±0.06	15.21 ±0.04	15.35 ±0.04	9.564	10.186
**CMDX-2 (SAXS)**	3.68	43.10 ±0.03	122.59 ±0.07	15.07 ±0.04	15.19 ±0.04	9.605	10.143
**CMDX-3 (SAXS)**	5.13	44.70 ±0.03	119.37 ±0.07	12.60 ±0.04	12.57 ±0.04	9.625	10.217
**CMDX-4 (SAXS)**	5.94	45.38 ±0.03	117.12 ±0.08	11.88 ±0.05	12.01 ±0.05	9.634	10.228
**CMDX-5 (SAXS)**	7.07	45.77 ±0.03	122.86 ±0.08	11.13 ±0.05	12.03 ±0.05	9.667	10.247
**CMDX-6 (SAXS)**	8.37	43.66 ±0.03	114.77 ±0.07	13.51 ±0.04	13.56 ±0.04	9.624	10.182
**CMDX-7 (SAXS)**	9.25	42.69 ±0.03	114.17 ±0.07	14.31 ±0.04	14.22 ±0.04	9.610	10.166
**CMDX-8 (SAXS)**	9.77	43.15 ±0.03	114.01 ±0.07	13.18 ±0.05	13.22 ±0.05	9.601	10.205
**CMDX-9 (SAXS)**	10.80	41.61 ±0.02	113.42 ±0.07	13.77 ±0.04	13.51 ±0.04	9.572	10.205
**CMDX-10 (SAXS)**	11.63	40.87 ±0.02	113.38 ±0.06	14.12 ±0.04	13.81 ±0.04	9.555	10.189
**MCDX-1 (SAXS)**	3.02	40.28 ±0.02	114.83 ±0.06	12.93 ±0.04	13.66 ±0.04	9.593	10.127
**MCDX-2 (SAXS)**	4.14	40.00 ±0.02	118.78 ±0.06	14.95 ±0.04	15.18 ±0.04	9.594	10.066
**MCDX-3 (SAXS)**	4.88	39.52 ±0.02	129.71 ±0.07	15.65 ±0.04	16.35 ±0.04	9.595	10.074
**MCDX-4 (SAXS)**	6.45	38.78 ±0.02	148.83 ±0.09	18.14 ±0.04	20.14 ±0.05	9.596	10.032
**MCDX-5 (SAXS)**	6.98	39.49 ±0.02	140.65 ±0.08	17.20 ±0.04	17.79 ±0.04	9.600	10.063
**MCDX-6 (SAXS)**	8.53	38.92 ±0.02	128.92 ±0.07	14.38 ±0.04	14.59 ±0.04	9.596	10.148
**MCDX-7 (SAXS)**	9.06	39.42 ±0.03	119.86 ±0.08	13.74 ±0.05	14.33 ±0.05	9.521	10.095
**MCDX-8 (SAXS)**	9.85	37.45 ±0.02	115.75 ±0.06	11.01 ±0.04	12.02 ±0.04	9.499	10.311
**MCDX-9 (SAXS)**	10.82	36.74 ±0.02	108.07 ±0.06	11.15 ±0.04	12.14 ±0.04	9.482	10.306
**MCDX-10 (SAXS)**	11.74	35.67 ±0.02	92.02 ±0.05	11.70 ±0.03	11.82 ±0.03	9.465	10.275

**RCMDX-1 (SAXS)**	2.80	35.85 ±0.04	106.6 ±0.1	16.76 ±0.07	17.05 ±0.07	9.581	10.617
**RCMDX-2 (SAXS)**	3.61	36.20 ±0.04	111.9 ±0.1	16.72 ±0.07	16.41 ±0.07	9.598	10.620
**RCMDX-3 (SAXS)**	5.83	38.00 ±0.05	147.30 ±0.2	15.66 ±0.09	17.46 ±0.1	9.692	10.678
**RCMDX-4 (SAXS)**	6.61	35.76 ±0.04	127.93 ±0.2	18.19 ±0.07	17.69 ±0.07	9.529	10.688
**RCMDX-5 (SAXS)**	7.65	36.32 ±0.04	115.0 ±0.1	16.55 ±0.07	16.62 ±0.07	9.533	10.763
**RCMDX-6 (SAXS)**	8.73	36.56 ±0.04	106.8 ±0.1	15.39 ±0.07	13.41 ±0.06	9.533	10.840
**RCMDX-7 (SAXS)**	9.25	36.84 ±0.05	94.4 ±0.1	16.50 ±0.08	15.32 ±0.07	9.648	10.632
**RCMDX-8 (SAXS)**	9.82	35.66 ±0.07	88.1 ±0.2	15.6 ±0.1	13.9 ±0.1	9.538	10.738
**RCMDX-9 (SAXS)**	11.05	35.16 ±0.04	91.6 ±0.1	16.82 ±0.07	15.57 ±0.07	9.592	10.618
**RCMDX-10 (SAXS)**	11.64	33.90 ±0.04	91.6 ±0.1	17.19 ±0.07	16.23 ±0.07	9.581	10.617

[a.] The theoretical X-ray SLD values of the respective hydrophobic segments and hydrophilic segments of the polypeptoid block copolymers ($C_1M_{19}D_5$) in the micelles are $SLD_{thero,D5}$ = 9.020 and $SLD_{thero,C1M19}$ = 10.729. The X-ray SLD of H_2O is 9.469. The SLD values of the micellar core and corona obtained from SAXS data analyses suggest a fuzzy micellar core-shell interface and solvent penetration and counter-ion association within the micelle.

Table S4. ζ-potential of respective CMDX, MCDX and RCMDX micelles in aqueous solution with different solution pHs.

Sample Name	pH [a]	NaCl (mM)	ζ-Potential (mV)
**CMDX-1**	2.84	60	2.9±0.3
**CMDX-2**	3.39	60	−3.3±0.5
**CMDX-3**	4.39	60	−7.0±0.2
**CMDX-4**	5.27	60	−11.6±0.5
**CMDX-5**	6.81	60	−14.5±0.9
**CMDX-6**	8.27	60	−14.6±0.7
**CMDX-7**	8.81	60	−15.2±0.5
**CMDX-8**	9.09	60	−12.9±0.4
**CMDX-9**	9.97	60	−17.2±0.6
**CMDX-10**	10.95	60	−17.2±0.3
**CMDX-11**	11.69	60	−15.8±1
**CMDX-12**	11.90	60	−14.8±0.6
**MCDX-1**	2.63	60	4.7±1

**MCDX-2**	2.90	60	4.3±0.7
**MCDX-3**	4.21	60	2.9±0.6
**MCDX-4**	5.40	60	−0.5±0.7
**MCDX-5**	6.49	60	−4.3±1
**MCDX-6**	8.93	60	−3.9±0.7
**MCDX-7**	9.65	60	−4.0±0.4
**MCDX-8**	11.27	60	−2.8±1.9
**MCDX-9**	11.53	60	−4.0±1
**RCMDX-1**	3.02	60	5.0±0.5
**RCMDX-2**	3.77	60	3.7±0.4
**RCMDX-3**	5.84	60	−0.6±0.3
**RCMDX-4**	6.56	60	−2.4±0.4
**RCMDX-5**	7.53	60	−4.0±1
**RCMDX-6**	8.53	60	−5.0±1
**RCMDX-7**	9.01	60	−5.6±0.3
**RCMDX-8**	9.54	60	−7.1±0.5
**RCMDX-9**	10.69	60	−7.5±0.6
**RCMDX-10**	11.23	60	−8.9 ±0.7

[a.] pH values of all micellar solutions in D_2O was obtained by using the following relation: pH = pH* ×0.929 + 0.42 where pH* is the direct reading of a H_2O-calibrated pH meter in a D_2O solution.[S7]

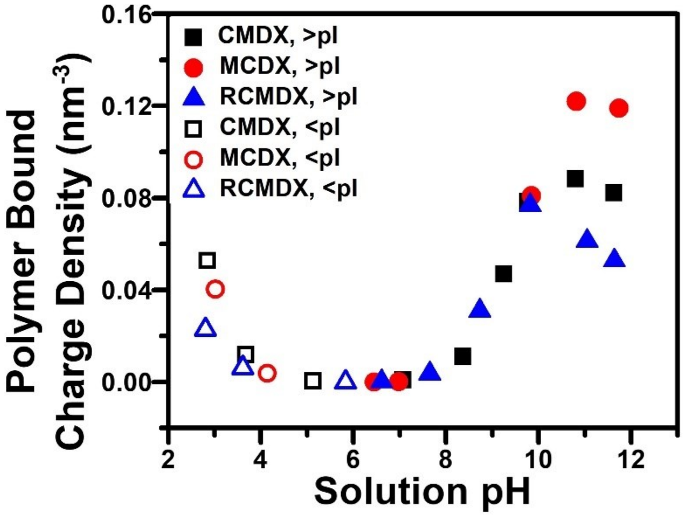

Figure S14. Estimated polymer bound charge density (Eq. S10) in the micellar corona at different solution pHs for CMDX (black squares), MCDX (red circles), and RCMDX (blue triangles).

References

S1. Heller, W. T.; Hetrick, J.; Bilheux, J.; Calvo, J. M. B.; Chen, W.-R.; DeBeer-Schmitt, L.; Do, C.; Doucet, M.; Fitzsimmons, M. R.; Godoy, W. F.; et al. drtsans: The data reduction toolkit for small-angle neutron scattering at Oak Ridge National Laboratory. *Software X* **2022**, *19*, 101101.

S2. Murnen, H. K.; Rosales, A. M.; Dobrynin, A. V.; Zuckermann, R. N.; Segalman, R. A. Persistence length of polyelectrolytes with precisely located charges. *Soft Matter* **2013**, *9* (1), 90-98.

S3. Rosales, A. M.; McCulloch, B. L.; Zuckermann, R. N.; Segalman, R. A. Tunable Phase Behavior of Polystyrene–Polypeptoid Block Copolymers. *Macromolecules* **2012**, *45* (15), 6027-6035.

S4. Sun, J.; Jiang, X.; Lund, R.; Downing, K. H.; Balsara, N. P.; Zuckermann, R. N. Self-assembly of crystalline nanotubes from monodisperse amphiphilic diblock copolypeptoid tiles. *Proc. Natl. Acad. Sci.* **2016**, *113* (15), 3954-3959.

S5. Kotlarchyk, M.; Chen, S.-H. Analysis of small angle neutron scattering spectra from polydisperse interacting colloids. *J. Chem. Phys.* **1983**, *79* (5), 2461-2469.

S6. Berr, S. S. Solvent Isotope Effects on Alkyltrimethylammonium Bromide Micelles as a Function of Alkyl Chain Length. *J. Phys. Chem.* **1987**, *91* (18), 4760-4765.

S7. Krezel, A.; Bal, W. A formula for correlating pKa values determined in D_2O and H_2O. *J. Inorg. Biochem.* **2004**, *98* (1), 161-166.
